# Supplementary material for: LSD1/PRMT6-targeting gene therapy to attenuate androgen receptor toxic gain-of-function ameliorates spinobulbar muscular atrophy phenotypes in flies and mice
Source: Nat Commun. 2023 Feb 6;14:603. doi: 10.1038/s41467-023-36186-9 (PMC9902531; doi:10.1038/s41467-023-36186-9)
Supplement: Supplementary file 1 — Supplementary Information [file 41467_2023_36186_MOESM1_ESM.pdf]

Supplementary Materials

List of supplementary material

Supplementary Tables

Supplementary Table 1. SBMA patient clinical information.
Supplementary Table 2. CRISPR-Cas9 guides used to silence human (h*LSD1*) and mouse (m*Lsd1*) *LSD1* and mouse/human *PRMT6 (*mh*PRMT6)*.
Supplementary Table 3. amiR primers targeting mouse and human *PRMT6* and *LSD1*.
Supplementary Table 4. RT-PCR primers.
Supplementary Table 5. ChIP primers.
Supplementary Table 6. Antibody list

Supplementary Figures

Supplementary Fig. 1. LSD1 and PRMT6 are overexpressed selectively in SBMA skeletal muscle.
Supplementary Fig. 2. LSD1 interacts with normal and polyQ-expanded AR.
Supplementary Fig. 3. Overlapping subcellular localization of endogenous LSD1 with normal and polyQ-expanded AR.
Supplementary Fig. 4. LSD1 is a co-activator of normal and polyQ-expanded AR.
Supplementary Fig. 5. LSD1 requires the AF-2 surface of AR and its catalytic activity to transactivate normal AR.
Supplementary Fig. 6. LSD1 and PRMT6 interact and synergistically transactivate normal and polyQ-expanded AR.
Supplementary Fig. 7. Efficacy of amiR target silencing in vivo.
Supplementary Fig. 8. Effect of amiR treatment on gene expression.

Supplementary Data

Supplementary Data 1. DEGs from RNAseq analysis of SBMA and WT mice treated with vehicle and amiR-*Lsd1/Prmt6*.

Supplementary Fig. 1

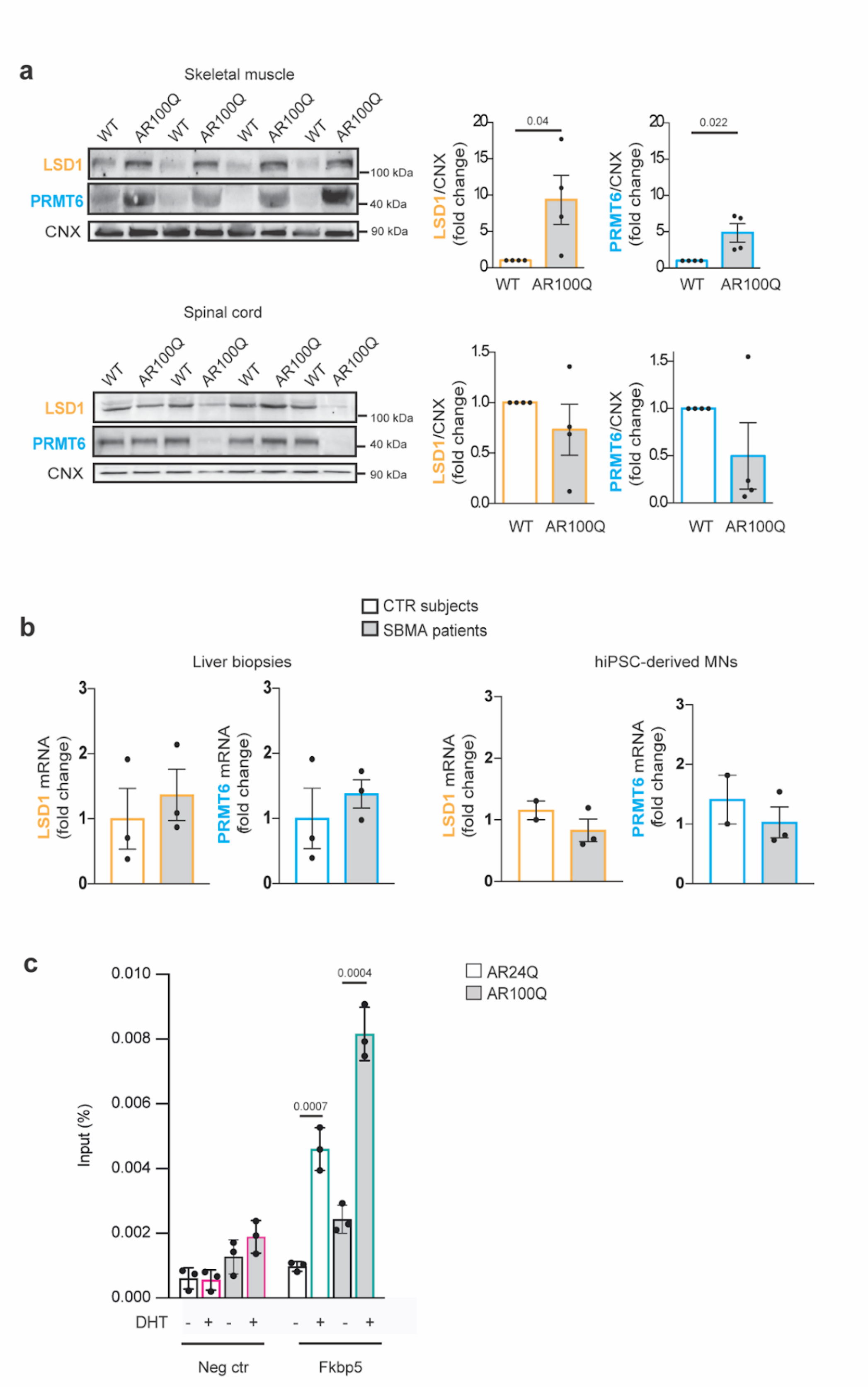

Supplementary Fig. 1. LSD1 and PRMT6 are overexpressed selectively in SBMA skeletal muscle.

- **a)** Western blot analysis of LSD1 and PRMT6 in the indicated tissues of 12-week-old WT and AR100Q mice (n = 4 mice/genotype). LSD1 and PRMT6 were detected with specific antibodies, and CNX was used as loading control. Molecular weight is indicated on the right.
- **b)** (Left) RT-PCR analysis of *LSD1* and *PRMT6* transcript levels normalized to *ACTIN* in the liver of SBMA patients and healthy controls (n = 3 control and 3 patient-derived biopsies).
  (Right) RT-PCR analysis of *LSD1* and *PRMT6* transcript levels normalized to *HPRT1* in patient-derived induced pluripotent stem cells (iPSCs) differentiated to motor neurons (n = 2 control and 3 SBMA patient-derived cell lines).
- **c)** Chromatin-immunoprecipitation assays of AR occupancy at a nonandrogen-responsive chromatin locus (negative control) and at the known AR-regulated gene, *Fkbp5* (positive control) in C2C12 myoblasts expressing AR24Q or AR100Q and treated with vehicle or DHT (10 nM, 12 h). Shown is one experiment representative of three technical replicates.

Graphs, mean ± sem, two-tailed student's t test. Source data are provided as a Source Data file.

Supplementary Fig. 2

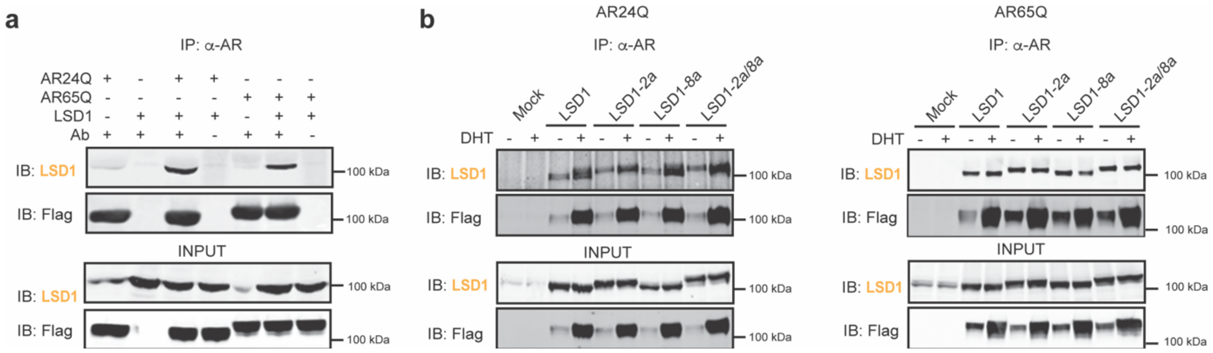

Supplementary Fig. 2. LSD1 interacts with normal and polyQ-expanded AR.

a,b) Immunoprecipitation (IP) and western blot (IB) analysis of Flag-tagged AR and LSD1 interaction in HEK293T cells (n = 3 biological replicates). AR was detected with anti-Flag antibody and LSD1 with a specific antibody. Molecular weight is indicated on the right. Source data are provided as a Source Data file.

Supplementary Fig. 3

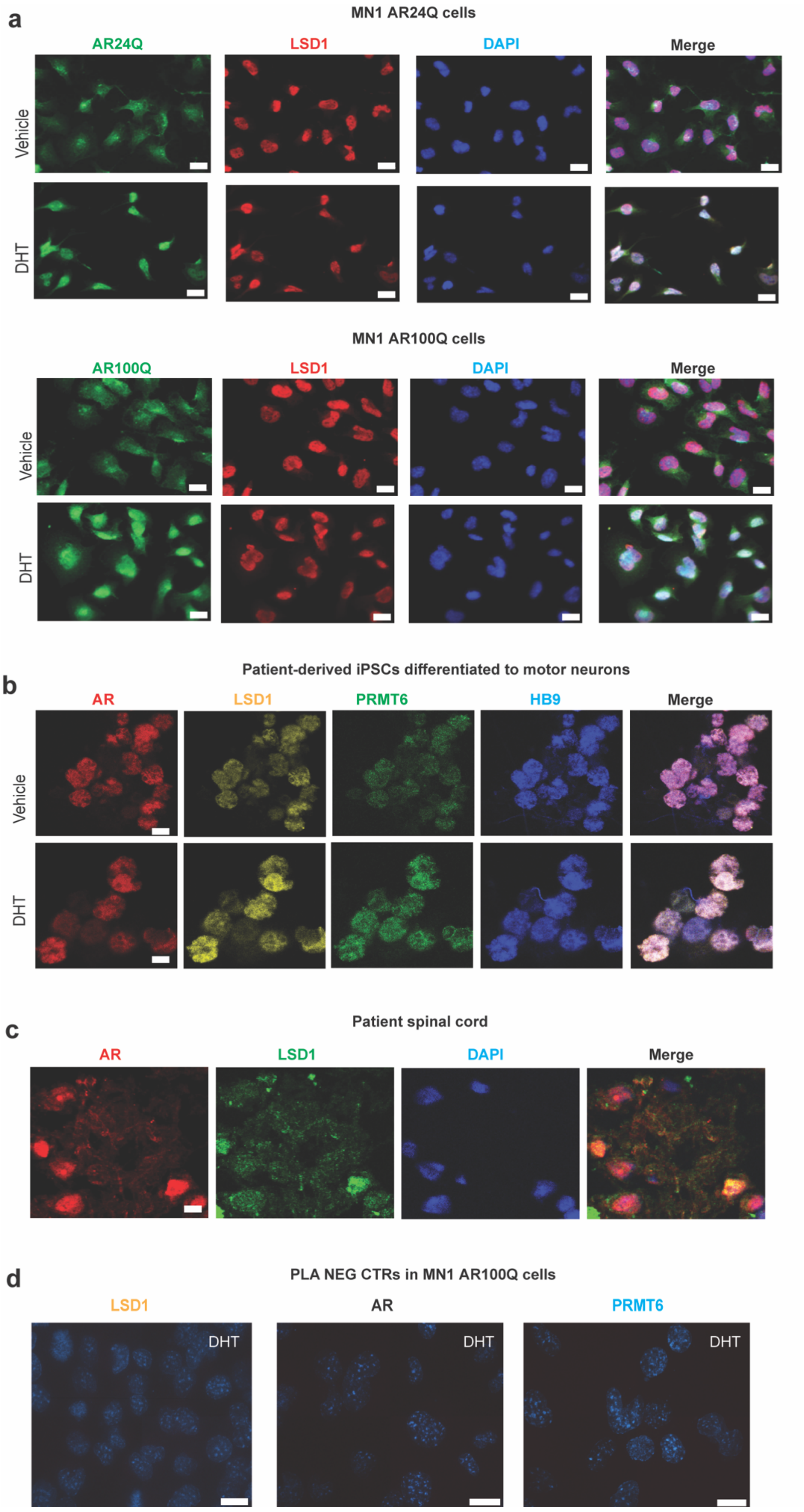

Supplementary Fig. 3. Overlapping subcellular localization of endogenous LSD1 with normal and polyQ-expanded AR.

a–c) Immunofluorescence analysis of the subcellular localization of normal and polyQ-expanded AR and LSD1 in MN1 cells treated with vehicle or DHT (10 nM, 16 h) (**a**), patient-derived iPSCs differentiated to motor neurons (**b**), and a spinal cord sample from a deceased SBMA patient (**c**). iPSCs also were stained for PRMT6 and motor neuron marker HB9. AR, PRMT6, LSD1, and HB9 were detected with specific antibodies, and nuclei were stained with DAPI. Shown are images representative of at least three independent experiments, except for panel c, which is the only spinal cord autopsy sample available. Scale bar = 20 µm (a) and 10 µm (b, c)

d) Negative controls for proximity ligation assay (PLA) in MN1 cells expressing AR100Q and treated with DHT (10 nM, 16 h). Only the antibody against LSD1 (left), AR (middle), or PRMT6 (right) was added to the reaction. Scale bar = 17 µm, n = 3 biological replicates.

Supplementary Fig. 4

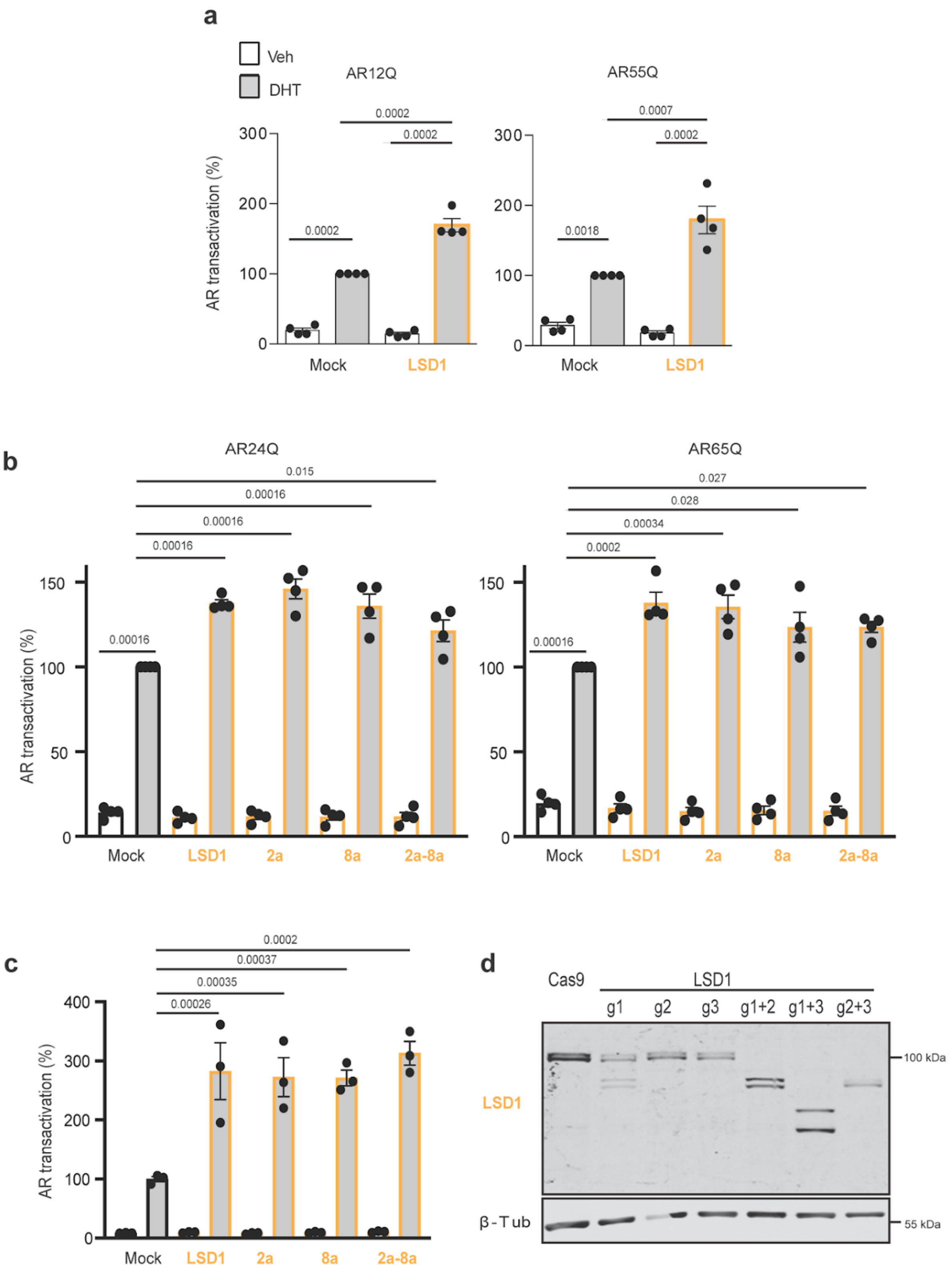

Supplementary Fig. 4. LSD1 is a co-activator of normal and polyQ-expanded AR.

a) Transcriptional assays in HEK293T cells expressing AR12Q or AR55Q driven by the elongation factor 1α promoter alone or together with LSD1 and treated with vehicle or DHT (10 nM, 16 h; n = 4 biological replicates).

b) Transcriptional assays in HEK293T cells expressing AR24Q or AR65Q alone or together with the indicated LSD1 isoforms and treated with vehicle or DHT (10 nM, 16 h; n = 4 biological replicates).

c) Transcriptional assays in MN1 cells expressing AR24Q alone or together with the indicated LSD1 isoforms and treated with vehicle or DHT (10 nM, 16 h; n = 3 biological replicates).

d) Western blot of LSD1 expression in HEK293T cells stably expressing Cas9 and the indicated guides targeting *LSD1*. Shown is one experiment representative of n = 7 biological replicates. LSD1 was detected with a specific antibody, and β-Tub was used as loading control.

Graphs, mean ± sem, two-way ANOVA followed by Tukey HSD test. Source data are provided as a Source Data file.

Supplementary Fig. 5

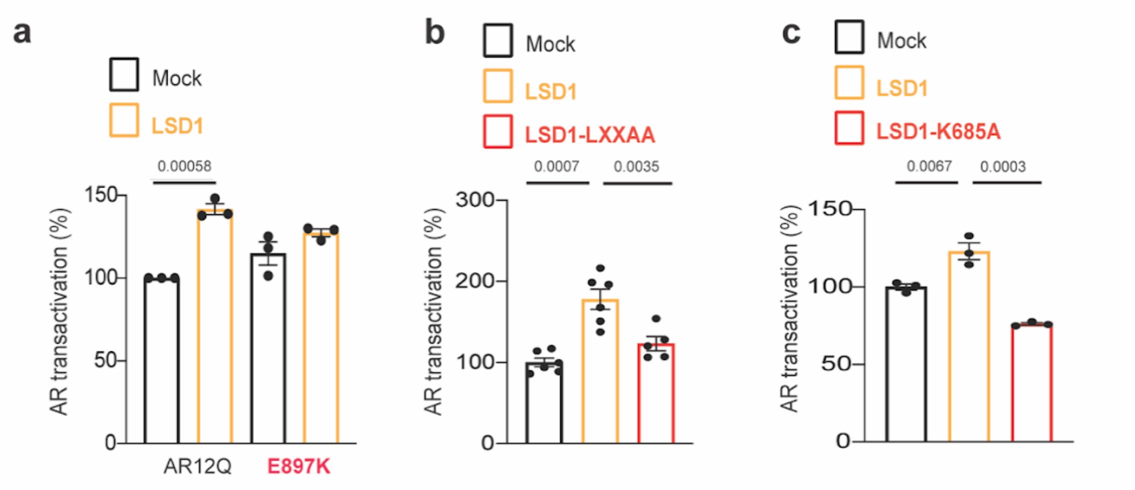

Supplementary Fig. 5. LSD1 requires the AF-2 surface of AR and its catalytic activity to transactivate normal AR.

- **a)** Transcriptional assay in HEK293T cells expressing AR12Q or AR12Q-E897K alone (mock) or together with LSD1. Cells were treated with DHT (10 nM, 16 h; n = 3 biological replicates).
- **b)** Transcriptional assay in HEK293T cells expressing AR24Q alone (mock) or with either LSD1 or LSD1-LXXAA. Cells were treated with DHT (10 nM, 16 h; n = 6 mock and LSD1 overexpression, and n = 5 LSD1-LXXAA overexpression biological replicates).
- **c)** Transcriptional assay in HEK293T cells expressing AR24Q alone (mock) or with LSD1 or the catalytic inactive mutant LSD1-K685A. Cells were treated with DHT (10 nM, 16 h; n = 3 biological replicates).

Graphs, mean ± sem, two-way ANOVA (a), one-way ANOVA (b, c) followed by Tukey HSD test. Source data are provided as a Source Data file.

Supplementary Fig. 6

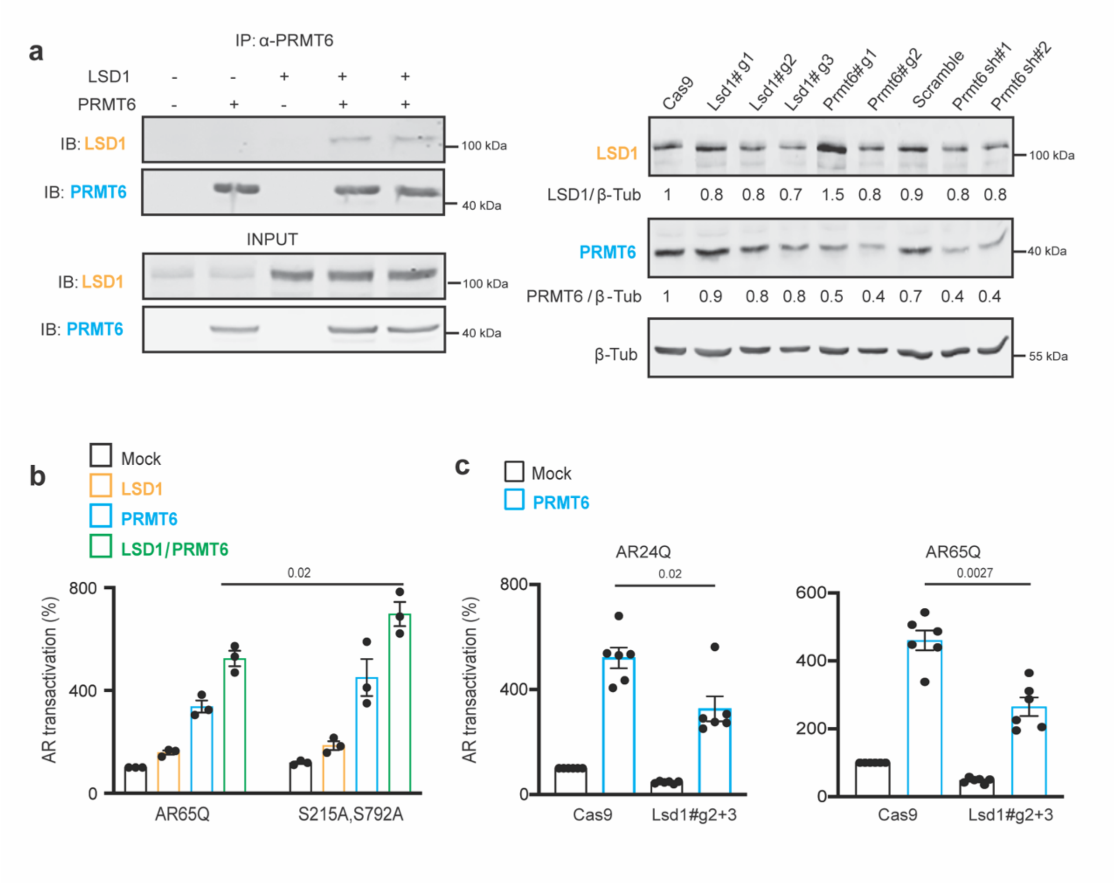

Supplementary Fig. 6. LSD1 and PRMT6 interact and synergistically transactivate normal and polyQ-expanded AR.

- **a)** (Left) Immunoprecipitation (IP) and immunoblot (IB) analysis of LSD1 and PRMT6 interaction in HEK293T cells expressing PRMT6 tagged with EGFP and LSD1. (Right) Western blot of LSD1 and PRMT6 in HEK293T cells transduced with lentiviral vectors to silence *LSD1* and *PRMT6* by CRISPR technology. Shown is one experiment representative of 3 (right) and 2 (left) biological replicates. Quantification is shown at the bottom. LSD1 and PRMT6 were detected with specific antibodies, and β-Tub was used as loading control.
- **b)** Transcriptional assay in HEK293T cells expressing AR65Q or AR65Q-S215A,S792A alone (mock) or with LSD1 and/or PRMT6. Cells were treated with DHT (10 nM, 16 h; n = 3 biological replicates).
- **c)** Transcriptional assay in HEK293T cells with and without silencing guides targeting *LSD1* and expressing AR24Q or AR65Q alone (mock) or with PRMT6. Cells were treated with DHT (10 nM, 16 h; n = 6 biological replicates).

Graphs, mean ± sem, two-way ANOVA followed by Tukey HSD test. Source data are provided as a Source Data file.

Supplementary Fig. 7

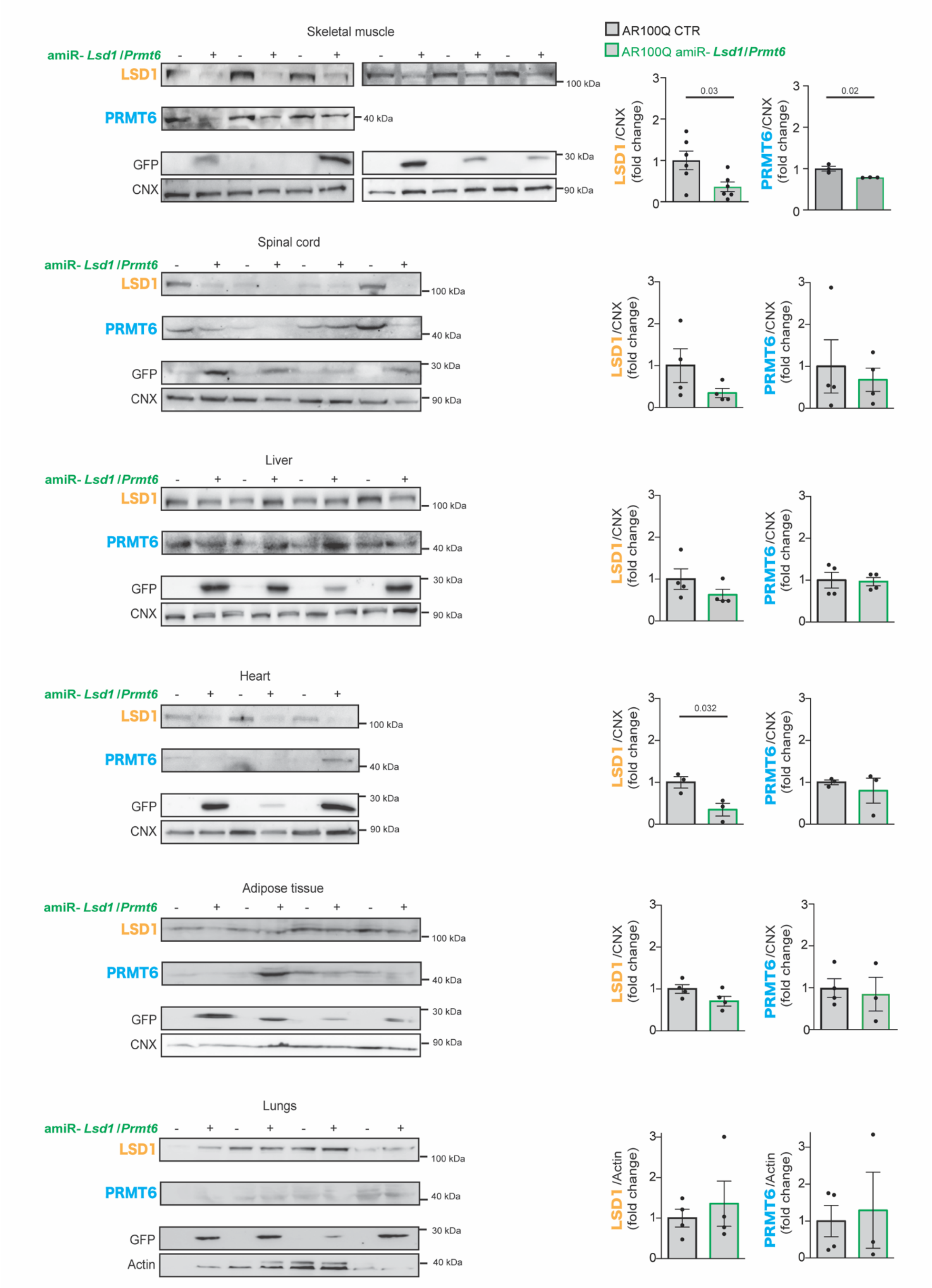

Supplementary Fig. 7. Efficacy of amiR target silencing in vivo.
Western blots and corresponding quantification in the indicated tissues of 13-week-old AR100Q mice treated with and without amiR-*Lsd1*/*Prmt6*.
Skeletal muscle LSD1 expression: n = 6 mice/treatment; PRMT6 expression: n = 3 mice/treatment; spinal cord, liver, adipose tissue and lungs: n = 4 mice/treatment; heart: n = 3 mice/treatment.
LSD1, PRMT6, and GFP were detected with specific antibodies, and CNX and actin were used as loading controls. Graphs, mean ± sem, two-tailed student's t test. Source data are provided as a Source Data file.

Supplementary Fig. 8

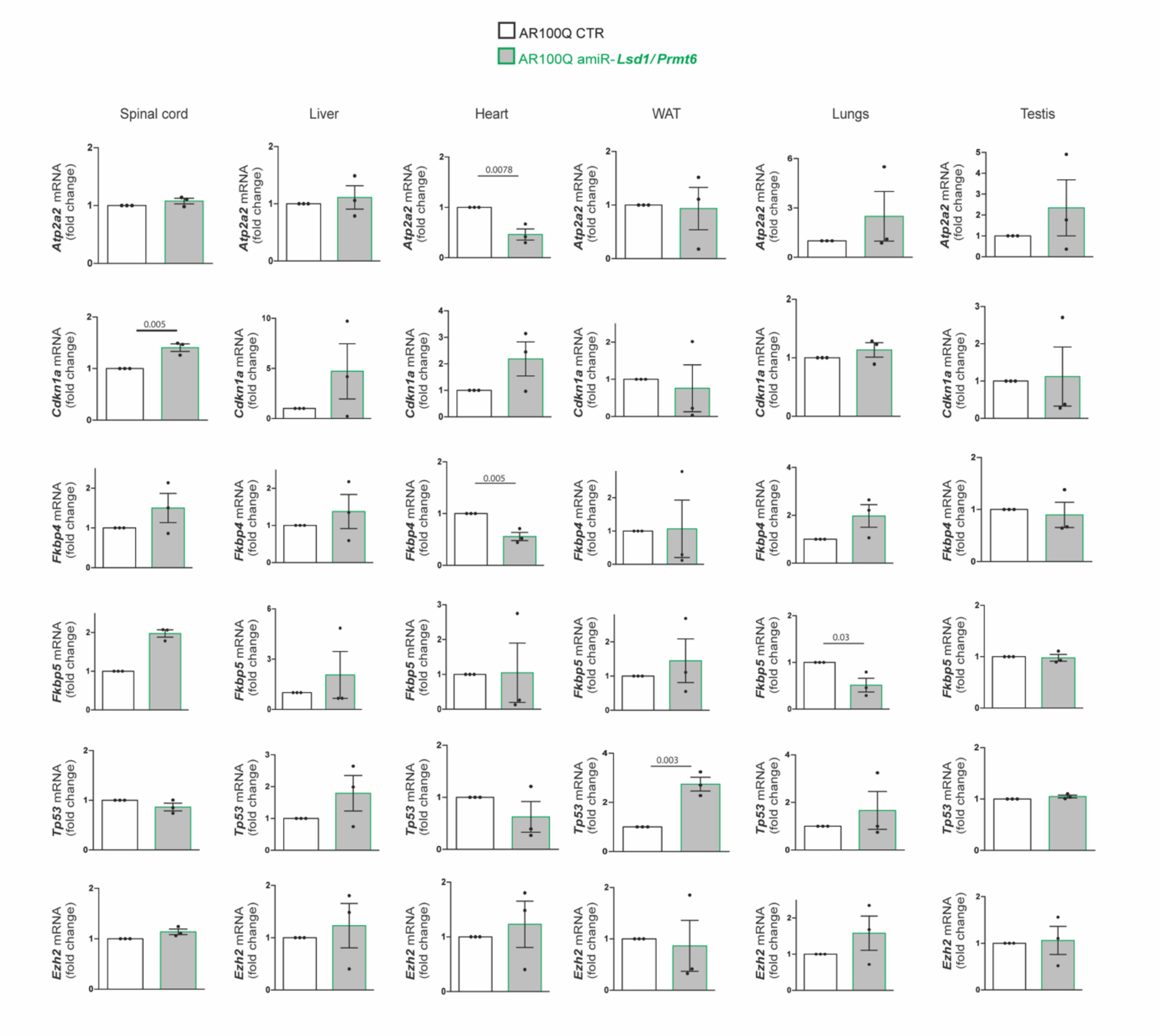

Supplementary Fig. 8. Effect of amiR treatment on gene expression.
RT-PCR analysis of LSD1, AR, and PRMT6 target genes in AR100Q mice treated with vehicle or amiR-*Lsd1*/*Prmt6* (n = 3 mice/genotype). Graphs, mean ± sem, two-tailed student's t test. Source data are provided as a Source Data file.

Supplementary Table 1. SBMA patient clinical information.

	Muscle	CAG repeat length
Control	Quadriceps femoris	/
Control	Vastus lateralis	/
Control	Vastus lateralis	/
Control	Vastus lateralis	/
Control	Vastus lateralis	/
SBMA	Quadriceps femoris	44
SBMA	Vastus lateralis	44
SBMA	Vastus lateralis	46
SBMA	Vastus lateralis	42
SBMA	Vastus lateralis	49
Control	Liver	/
Control	Liver	/
Control	Liver	/
SBMA	Liver	41
SBMA	Liver	46
SBMA	Liver	46

Supplementary Table 2. CRISPR-Cas9 guides used to silence human (h*LSD1*) and mouse (m*Lsd1*) *LSD1* and mouse/human *PRMT6 (*mh*PRMT6)*.

mh*PRMT6*-gRNA1-For	caccgAAAAGAAAGCTTGAGTCGG
mh*PRMT6*-gRNA1-Rev	aaacCCGACTCAAGCTTTCTTTTc
mh*PRMT6*-gRNA2-For	caccgCATCTGGCAACAGGCCCgGG
mh*PRMT6*-gRNA2-Rev	aaacCCcGGGCCTGTTGCCAGATGc
h*LSD1*-gRNA1-For	caccgTGGAATAGCAGAGACTCCGG
h*LSD1*-gRNA1-Rev	aaacCCGGAGTCTCTGCTATTCCAc
h*LSD1*-gRNA2-For	caccGCAGACCCAGGCACGACAGT
h*LSD1*-gRNA2-Rev	aaacACTGTCGTGCCTGGGTCTGC
h*LSD1*-gRNA3-For	caccgTGAGAAGTCATCCGGTCATG
h*LSD1*-gRNA3-Rev	aaacCATGACCGGATGACTTCTCAc
m*Lsd1*-gRNA4-For	caccgTGAGAGGTCATTCGGTCATG
m*Lsd1*-gRNA4-Rev	caccgTGAGAGGTCATTCGGTCATG

Supplementary Table 3. amiR primers targeting mouse and human *PRMT6* and *LSD1*

	**mouse PRMT6**	
	START=sense target sequence in the CDS of hPRMT6	
AmiR_1	ACCAGCTGTACTACGAGTGCT	TOP OLIGO (5’-3’)
		TGCTGAGCACTCGTAGTACAGCTGGTGTTTTGGCCACTGACTGACACCAGCTGCTACGAGTGCT
		BOTTOM (5’-3’)
		CCTGAGCACTCGTAGCAGCTGGTGTCAGTCAGTGGCCAAAACACCAGCTGTACTACGAGTGCTC
AmiR_2	TGTACTACGAGTGCTACTCCG	TOP OLIGO (5’-3’)
		TGCTGCGGAGTAGCACTCGTAGTACAGTTTTGGCCACTGACTGACTGTACTACGTGCTACTCCG
		BOTTOM (5’-3’)
		CCTGCGGAGTAGCACGTAGTACAGTCAGTCAGTGGCCAAAACTGTACTACGAGTGCTACTCCGC
AmiR_3	CTACCGCTTAGGCATCCTGAA	TOP OLIGO (5’-3’)
		TGCTGTTCAGGATGCCTAAGCGGTAGGTTTTGGCCACTGACTGACCTACCGCTGGCATCCTGAA
		BOTTOM (5’-3’)
		CCTGTTCAGGATGCCAGCGGTAGGTCAGTCAGTGGCCAAAACCTACCGCTTAGGCATCCTGAAC
AmiR_4	ACCGCTTAGGCATCCTGAAGA	TOP OLIGO (5’-3’)
		TGCTGTCTTCAGGATGCCTAAGCGGTGTTTTGGCCACTGACTGACACCGCTTACATCCTGAAGA
		BOTTOM (5’-3’)
		CCTGTCTTCAGGATGTAAGCGGTGTCAGTCAGTGGCCAAAACACCGCTTAGGCATCCTGAAGAC
AmiR_5	TCTGCGCATACTTCTGCGCTA	TOP OLIGO (5’-3’)
		TGCTGTAGCGCAGAAGTATGCGCAGAGTTTTGGCCACTGACTGACTCTGCGCACTTCTGCGCTA
		BOTTOM (5’-3’)
		CCTGTAGCGCAGAAGTGCGCAGAGTCAGTCAGTGGCCAAAACTCTGCGCATACTTCTGCGCTAC
AmiR_6	AATCAGACCATGTTGCCTTTC	TOP OLIGO (5’-3’)
		TGCTGAATCAGACCATGTTGCCTTTCGTTTTGGCCACTGACTGACGAAAGGCAATGGTCTGATT
		BOTTOM (5’-3’)
		CCTGAATCAGACCATTGCCTTTCGTCAGTCAGTGGCCAAAACGAAAGGCAACATGGTCTGATTC
AmiR_7	TATTAGATTAGAGTGCTCGCG	TOP OLIGO (5’-3’)
		TGCTGTATTAGATTAGAGTGCTCGCGGTTTTGGCCACTGACTGACCGCGAGCACTAATCTAATA
		BOTTOM (5’-3’)
		CCTGTATTAGATTAGTGCTCGCGGTCAGTCAGTGGCCAAAACCGCGAGCACTCTAATCTAATAC

**mouse LSD1**		
	START=sense target sequence in the CDS of hLSD1	
AmiR_ 1	CCAAGGTAGAATACAGAGAAA	TOP OLIGO (5’-3’)
		TGCTGTTTCTCTGTATTCTACCTTGGGTTTTGGCCACTGACTGACCCAAGGTAATACAGAGAAA
		BOTTOM (5’-3’)
		CCTGTTTCTCTGTATTACCTTGGGTCAGTCAGTGGCCAAAACCCAAGGTAGAATACAGAGAAAC
AmiR_ 2	TGTCGTCAGCAAACAAGTAAA	TOP OLIGO (5’-3’)
		TGCTGTTTACTTGTTTGCTGACGACAGTTTTGGCCACTGACTGACTGTCGTCAAAACAAGTAAA
		BOTTOM (5’-3’)
		CCTGTTTACTTGTTTTGACGACAGTCAGTCAGTGGCCAAAACTGTCGTCAGCAAACAAGTAAAC
AmiR_ 3	GTGATGTATACCTCTCATCAA	TOP OLIGO (5’-3’)
		TGCTGTTGATGAGAGGTATACATCACGTTTTGGCCACTGACTGACGTGATGTACCTCTCATCAA
		BOTTOM (5’-3’)
		CCTGTTGATGAGAGGTACATCACGTCAGTCAGTGGCCAAAACGTGATGTATACCTCTCATCAAC

human PRMT6		
	START=sense target sequence in the CDS of hPRMT6	
AmiR_1	CAACAACGGATACAGCGTGCT	TOP OLIGO (5’-3’)
		TGCTGAGCACGCTGTATCCGTTGTTGGTTTTGGCCACTGACTGACCAACAACGTACAGCGTGCT
		BOTTOM (5’-3’)
		CCTGAGCACGCTGTACGTTGTTGGTCAGTCAGTGGCCAAAACCAACAACGGATACAGCGTGCTC
AmiR_2	CGGATACAGCGTGCTTATTAT	TOP OLIGO (5’-3’)
		TGCTGATAATAAGCACGCTGTATCCGGTTTTGGCCACTGACTGACCGGATACAGTGCTTATTAT
		BOTTOM (5’-3’)
		CCTGATAATAAGCACTGTATCCGGTCAGTCAGTGGCCAAAACCGGATACAGCGTGCTTATTATC
AmiR_3	TCCCTGACACCTCACACCTTA	TOP OLIGO (5’-3’)
		TGCTGTAAGGTGTGAGGTGTCAGGGAGTTTTGGCCACTGACTGACTCCCTGACCTCACACCTTA
		BOTTOM (5’-3’)
		CCTGTAAGGTGTGAGGTCAGGGAGTCAGTCAGTGGCCAAAACTCCCTGACACCTCACACCTTAC

human LSD1		
	START=sense target sequence in the CDS of hLSD1	
AmiR_1	GTCCACCGAGTTCACAGTTAT	TOP OLIGO (5’-3’)
		TGCTGATAACTGTGAACTCGGTGGACGTTTTGGCCACTGACTGACGTCCACCGTTCACAGTTAT
		BOTTOM (5’-3’)
		CCTGATAACTGTGAACGGTGGACGTCAGTCAGTGGCCAAAACGTCCACCGAGTTCACAGTTATC
AmiR_2	TCACAGTTATTTAGAGCGTCA	TOP OLIGO (5’-3’)
		TGCTGTGACGCTCTAAATAACTGTGAGTTTTGGCCACTGACTGACTCACAGTTTTAGAGCGTCA
		BOTTOM (5’-3’)
		CCTGTGACGCTCTAAAACTGTGAGTCAGTCAGTGGCCAAAACTCACAGTTATTTAGAGCGTCAC
AmiR_3	TGACACTGTCAAGGTTCCTAA	TOP OLIGO (5’-3’)
		TGCTGTTAGGAACCTTGACAGTGTCAGTTTTGGCCACTGACTGACTGACACTGAAGGTTCCTAA
		BOTTOM (5’-3’)
		CCTGTTAGGAACCTTCAGTGTCAGTCAGTCAGTGGCCAAAACTGACACTGTCAAGGTTCCTAAC

Supplementary Table 4. RT-PCR primers.

MOUSE PRIMERS

Gene	Forward	Reverse
Prmt6	AGTCCATGCTGAGCTCCGT	TCCATGCAGCTCATATCCA
Lsd1	TGGAACTGGCCAAGATCAAG	GCTTCTAGCAACCGGTTAAATTC
Actin	GACAGGATGCAGAAGGAGATTACTG	CTCAGGAGGAGCAATGATCTTGAT
AR	GCCCGAATGCAAAGGTCTT	TGGCGTAACCTCCCTTGAAA
Musk	ATCACCACGCCTCTTGAAAC	TGTCTTCCACGCTCAGAATC
Myog	CTTGCTCAGCTCCCTCAAC	TGGGAGTTGCATTCACTGG
Ncam	ACAATGCTGCGAACTAAGGA	TGCCACTTGACACAGGA
Cdkn1a	AGGACCACGTGGCCTTGTC	TTTTCGGCCCTGAGATGTTC
Fkbp4	GACCGAGTCTTTGTCCACTACAC	ATCCCAAGCCTTGATGACCTCC
Fkbp5	AAAGGACAATGACTACTGATGAGG	CTGACAACATCCCTTTGTAGTGGAC
Tp53	GGGACGGGACAGCTTTGAG	AGGACTTCCTTTTTGCGGAAA
Ezh2	CATACGCTCTTCTGTCGACGATG	ACACTGTGGTCCACAAGGCTTG

HUMAN PRIMERS

Gene	Forward	Reverse
PRMT6	GAGTGCTACTCGGACGTTTC	AGGATACCCAGGCGGTAG
LSD1	AGTGAGCCTGAAGAACCATC	TCTGTTGTGGTCCACTGATAAT
AR	TGGGAGAGAGACAGCTTGTA	AGTACTGAATGACAGCCATCTG
ATPA2	TACCGAATTGAAGGGTCTTTCT	CGACATTGACTTTCTGTCACG
CDKN1A	AAGACCATGTGGACCTGTCA	GGCTTCCTCTTGGAGAAGAT
ACTIN	GGACTTCGAGCAAGAGATGG	AGCACTGTGTTGGCGTACAG

Supplementary Table 5. ChIP primers.

Primer	***5′ - 3′***
Neg_F	TCCCTGTGCCCAAAGAGTAG
Neg_R	CCCTGAAGAATGAGGACGAG
*Lsd1*_promoter_ARE_F	CAGTCTGGCCTACAAAGTGAG
*Lsd1*_promoter_ARE_R	GATTGGTTTTTCCGAGACAGG
*Prmt6*_promoter_ARE_F	ACTTGTCATGGCGTTAGAGG
*Prmt6*_promoter_ARE_R	GATTCGTAGTCCGCTTCCTG

Supplementary Table 6. Antibodies list

Antibody	Source	identifier	dilution
Anti PRMT6	Bethyl	A300-929A	1:2000 (PLA, IP and WB) and 1:200 (IF)
Anti PRMT6	Abcam	Ab151191	1:2000 (PLA)
Anti PRMT6	SantaCruz,	sc-55702(Q16)	1:50 (IF)
Anti PRMT6	Proteintech	15395-1-AP	1:1000 (WB and IP)
Anti LSD1	Abcam	ab17721	1:2000 (PLA), 1:200 (IF), 1:1000 (WB and IP)
Anti AR	Santa Cruz Biotechnology	441, sc-7305	1:2000 (PLA), 1:1000 (WB and IP)
Anti AR	GeneTex	GTX22742	1:50 (IF)
Anti AR	Santa Cruz Biotechnology	H280, sc-13062	1:1000 (WB and IP), 1:200 (IF)
Anti-HB9	Developmental Studies Hybridoma Bank	81.5C10	1: 100 (IF)
Anti FLAG	Sigma	7425	1:1000 (WB and IP)
Anti GFP	Roche	11814460001	1:1000 (WB)
Anti Calnexin	Enzo	ADI-SPA-860	1:2500 (WB)
Anti Tubulin	Sigma	T7816	1:10000 (WB)
Anti H3K4me2	Abcam	Ab7766	1:1000 (WB)
Anti-H3	Abcam	Ab1791	1:1000 (WB)
Donkey anti-Goat 647	Invitrogen	A-21447	1:2000 (IF)
Donkey anti-Rat 555	Invitrogen	A-78945	1:2000 (IF)
Donkey anti-Rabbit 488	Invitrogen	A-21206	1:2000 (IF)
Donkey anti-mouse 405	Invitrogen	A48257	1:2000 (IF)

Goat anti-rabbit HRP	Biorad	1706515	1:5000 (WB)
Goat anti-mouse HRP	Biorad	1706516	1:5000 (WB)

All the information related to the antibodies used in this paper are listed also in the Methods section and in the Reporting Summary.
