# Supplementary material for: LSD1/PRMT6-targeting gene therapy to attenuate androgen receptor toxic gain-of-function ameliorates spinobulbar muscular atrophy phenotypes in flies and mice
Source: Nat Commun. 2023 Feb 6;14:603. doi: 10.1038/s41467-023-36186-9 (PMC9902531; doi:10.1038/s41467-023-36186-9)
Supplement: Supplementary file 3 — Description of Additional Supplementary Files [file 41467_2023_36186_MOESM3_ESM.pdf]

Supplementary Data Legends

Supplementary Data 1. Differentially expressed genes between non-treated (AR100Q) and treated (amiR-*Lsd1*/*Prmt6*) SBMA mice and WT controls. We defined a set of "rescued genes" as genes showing a significant but opposite direction in AR100Q vs WT and amiR-*Lsd1/Prmt6* vs AR100Q differential expression analyses.
