# Supplementary material for: LSD1/PRMT6-targeting gene therapy to attenuate androgen receptor toxic gain-of-function ameliorates spinobulbar muscular atrophy phenotypes in flies and mice
Source: Nat Commun. 2023 Feb 6;14:603. doi: 10.1038/s41467-023-36186-9 (PMC9902531; doi:10.1038/s41467-023-36186-9)

Uncropped figures

Nature Communications

Uncropped fig.1b

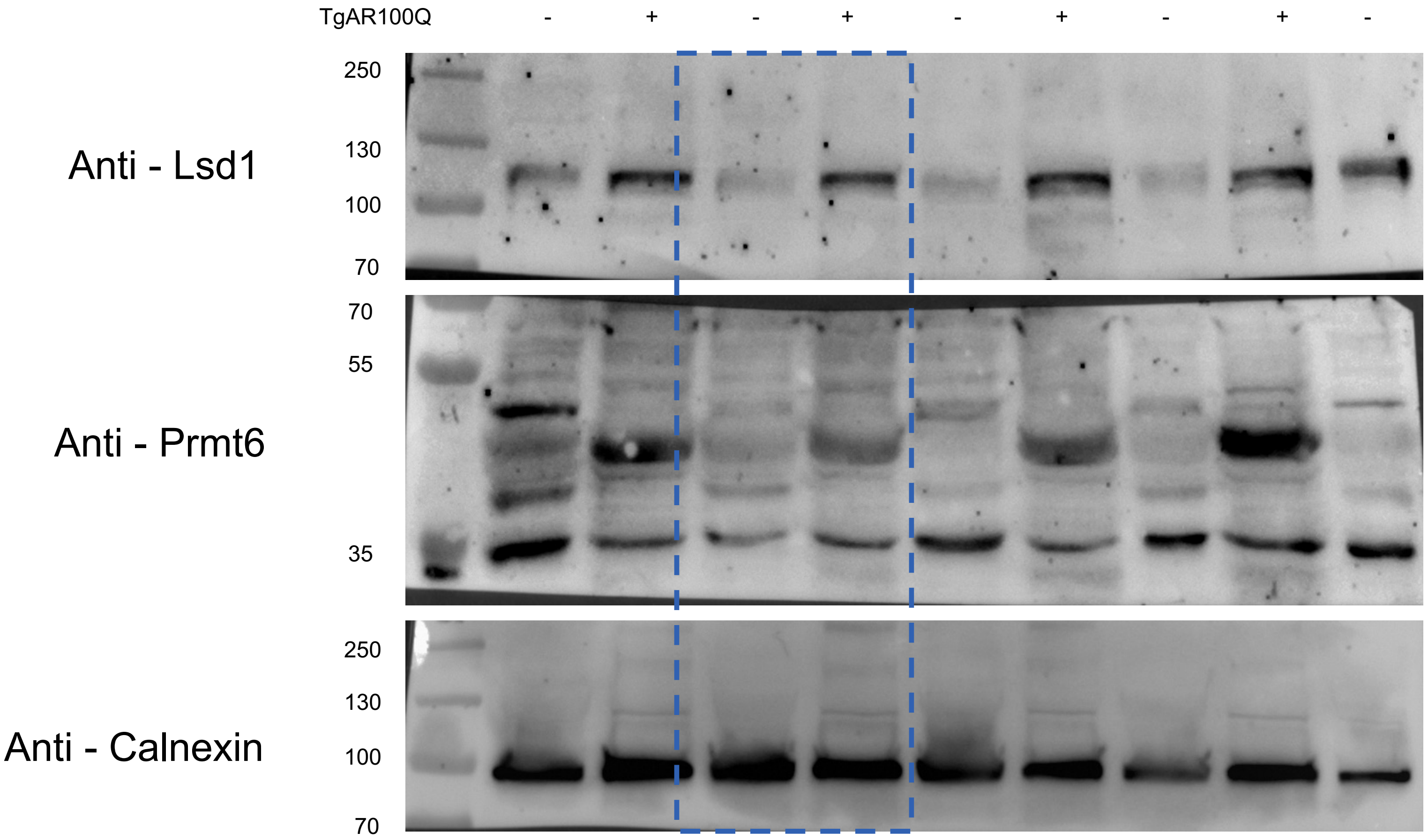

Uncropped fig. 2d

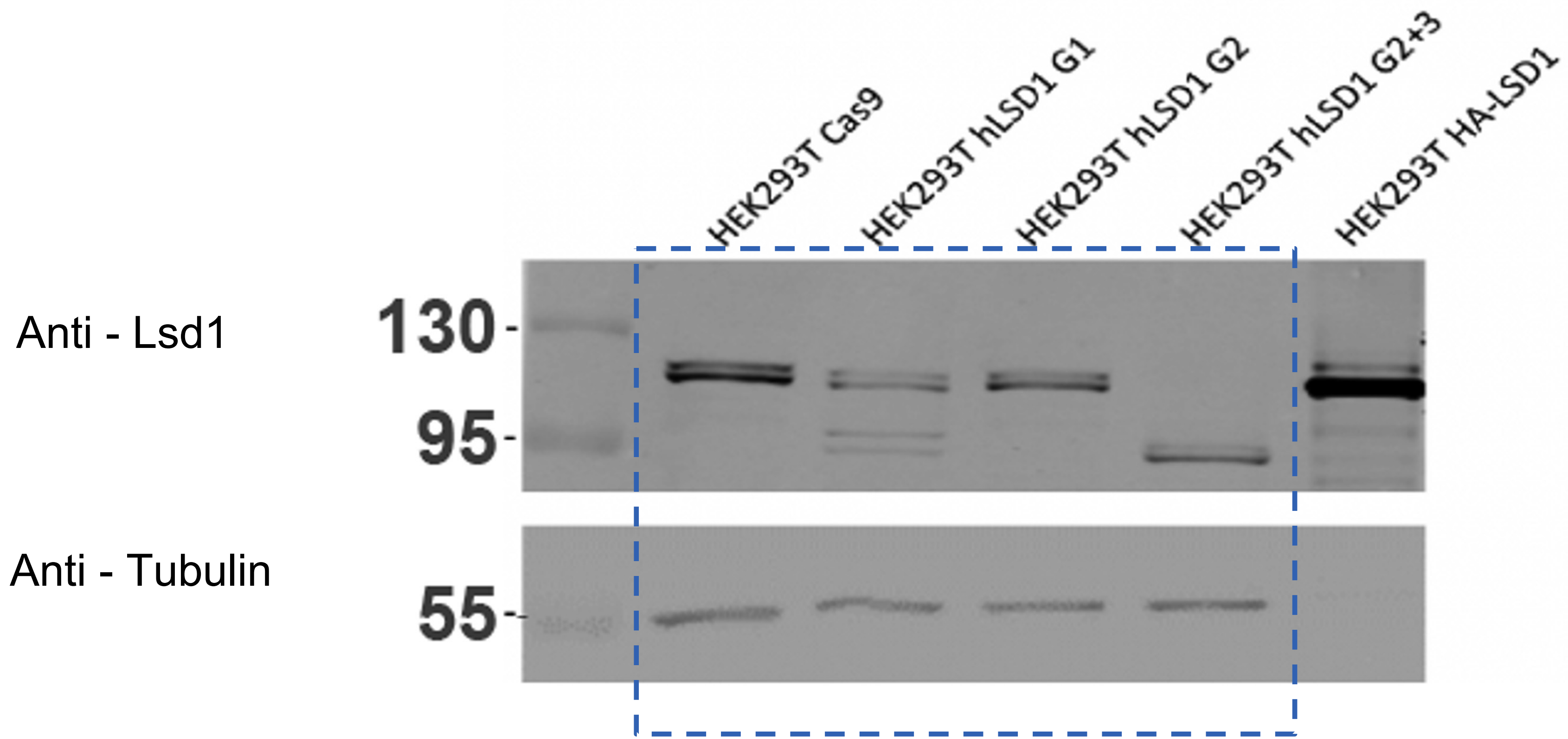

Uncropped fig. 2e

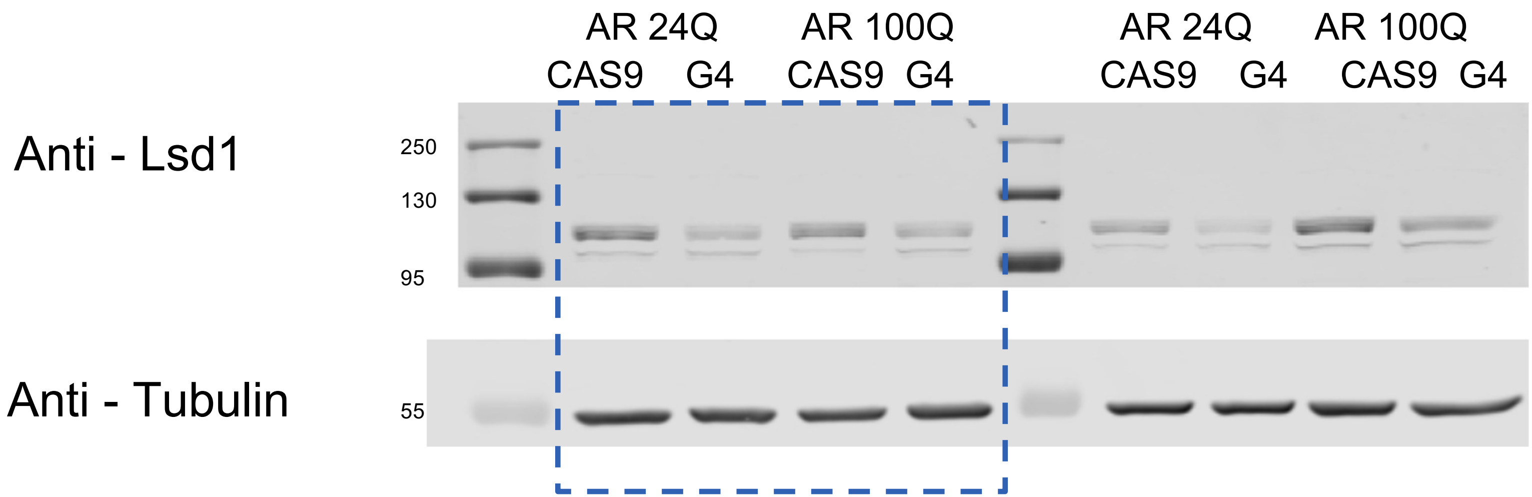

Uncropped fig. 3c

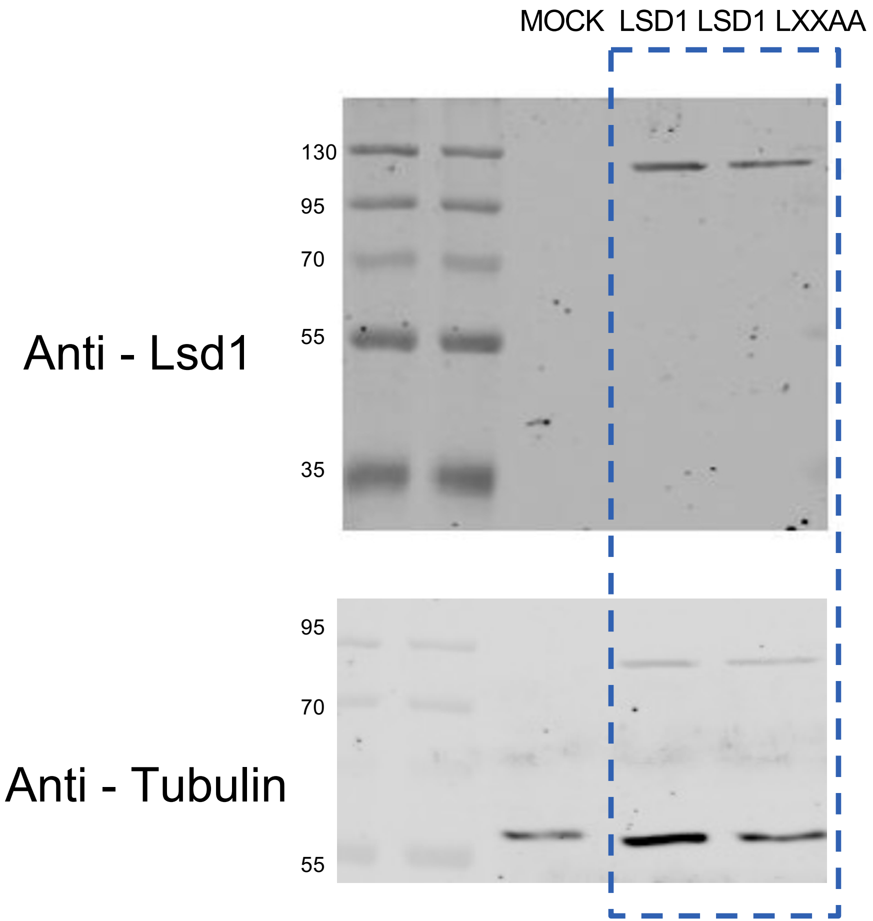

Uncropped fig. 3f

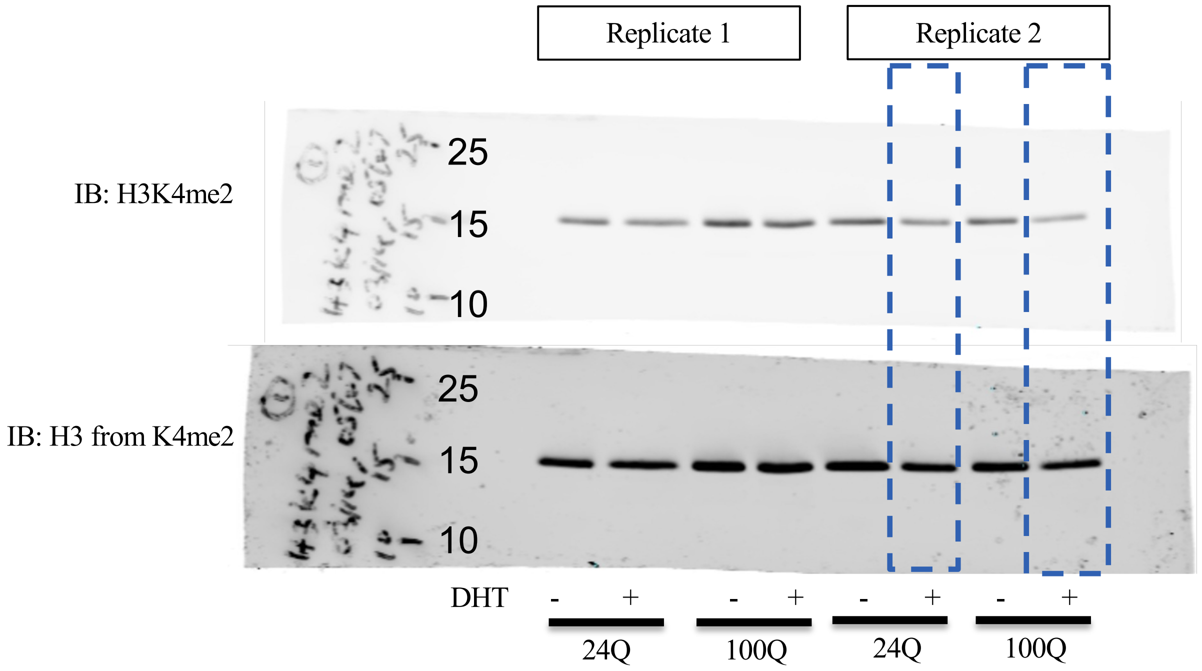

Uncropped fig. 4b (left)

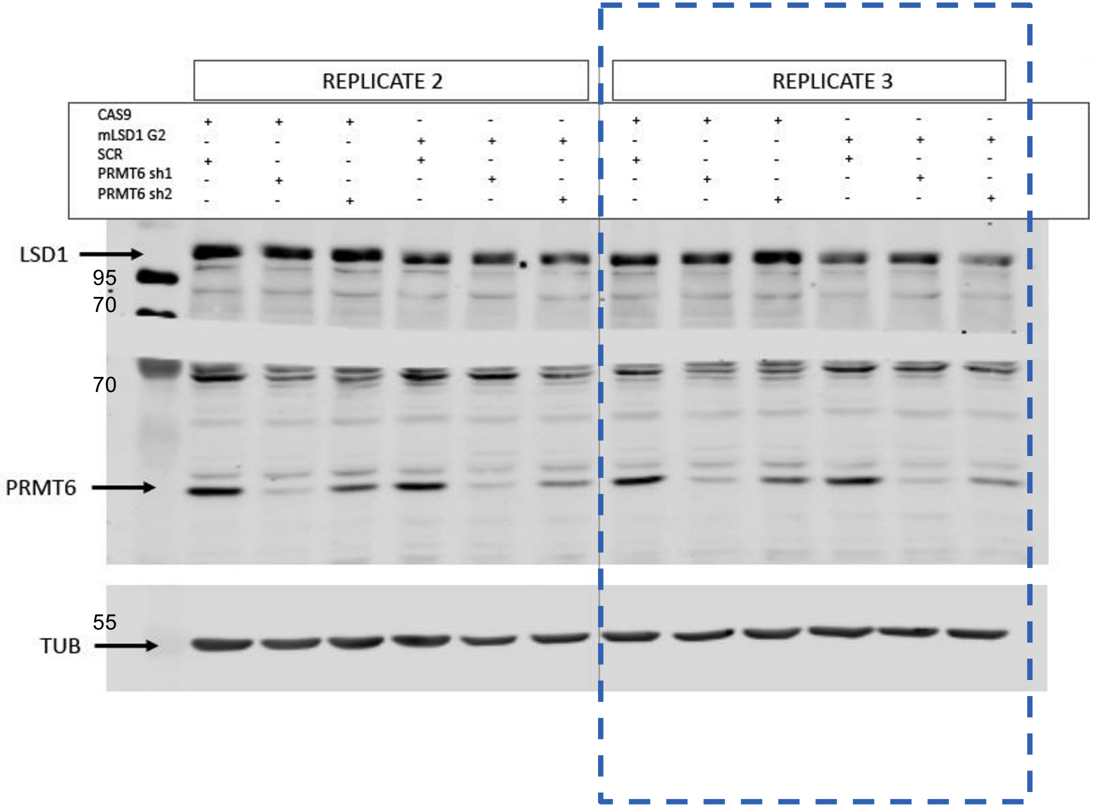

Uncropped fig.4b (right)

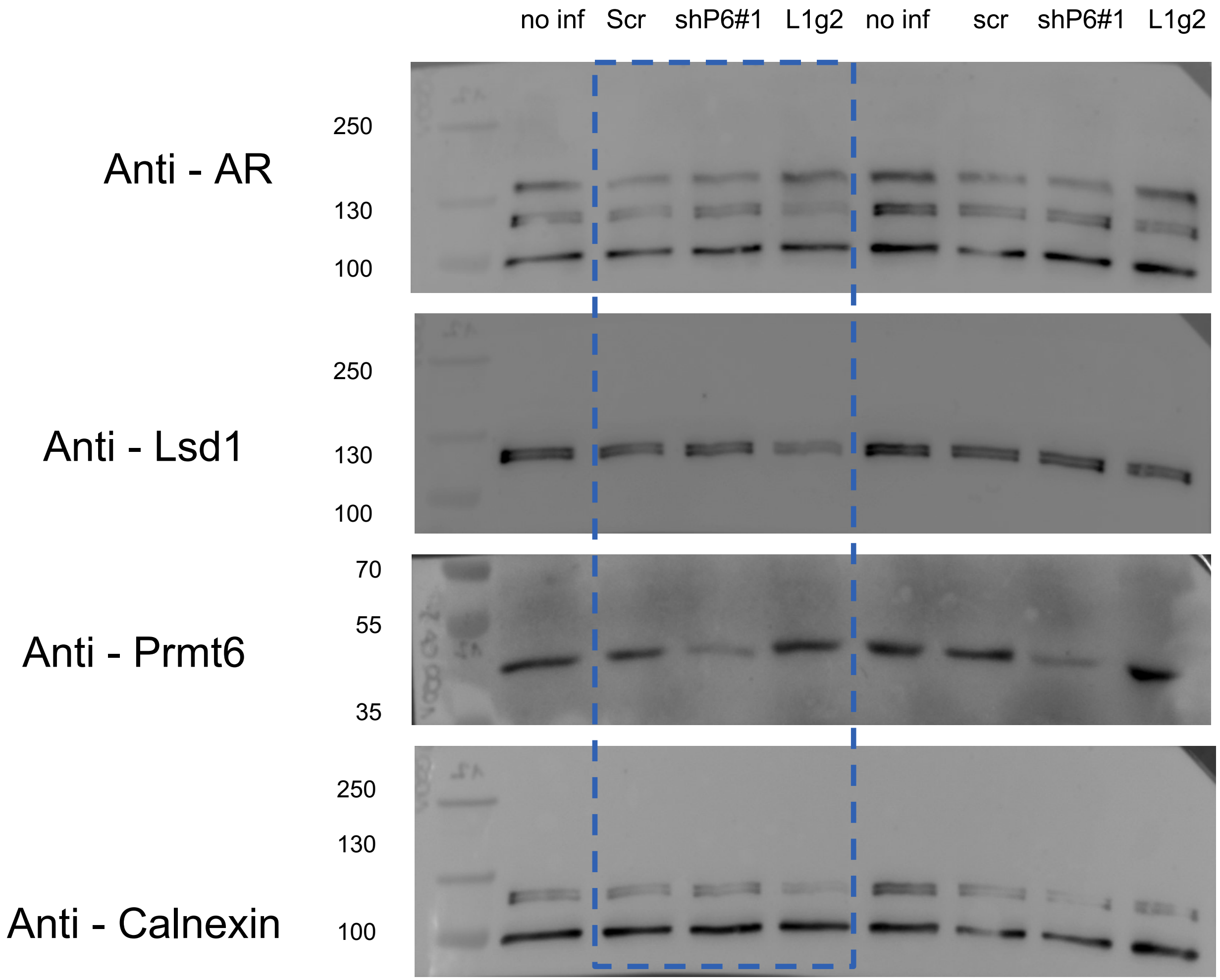

Uncropped fig. 4c IP MUSCLE

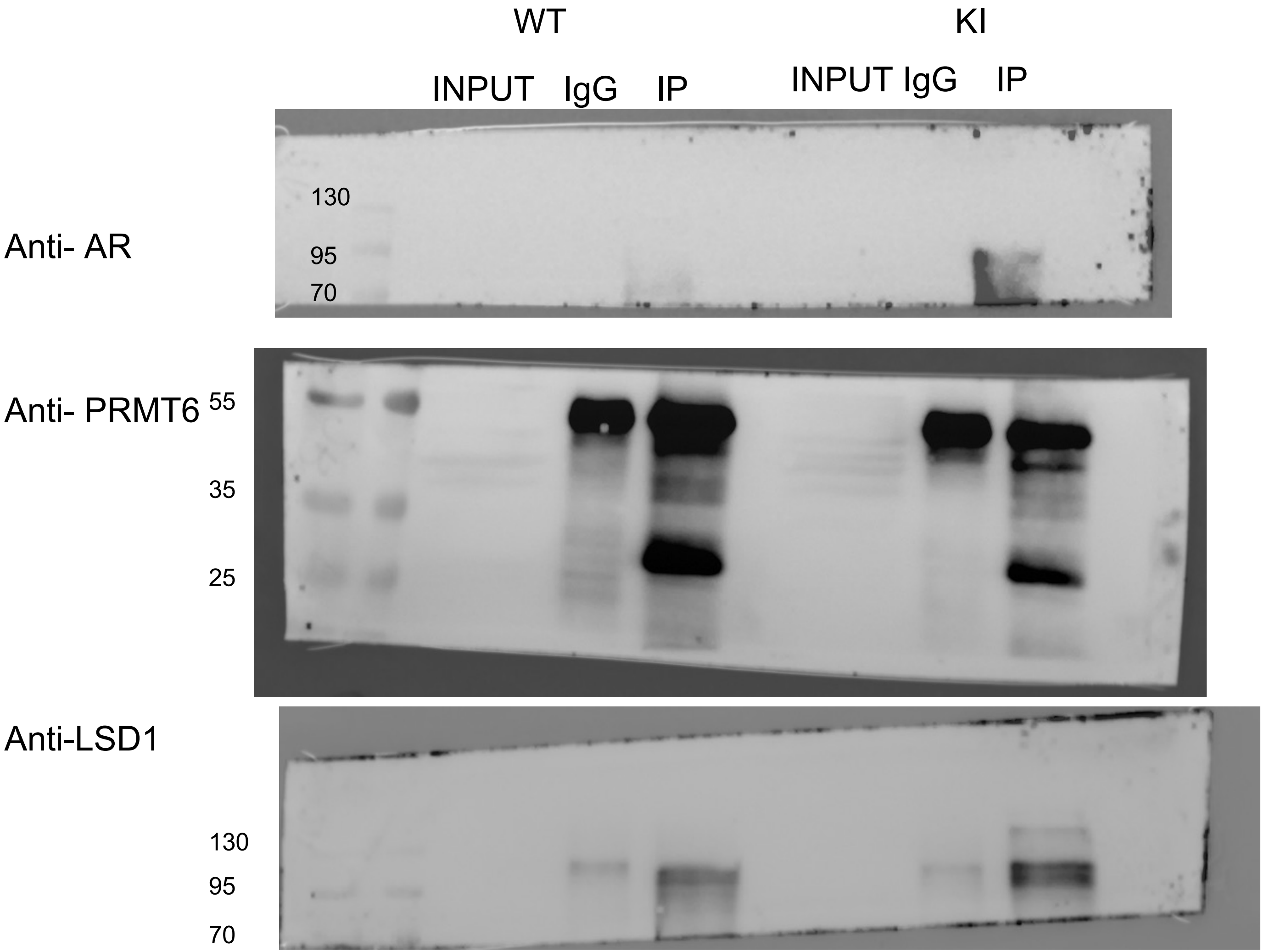

Uncropped fig. 4e

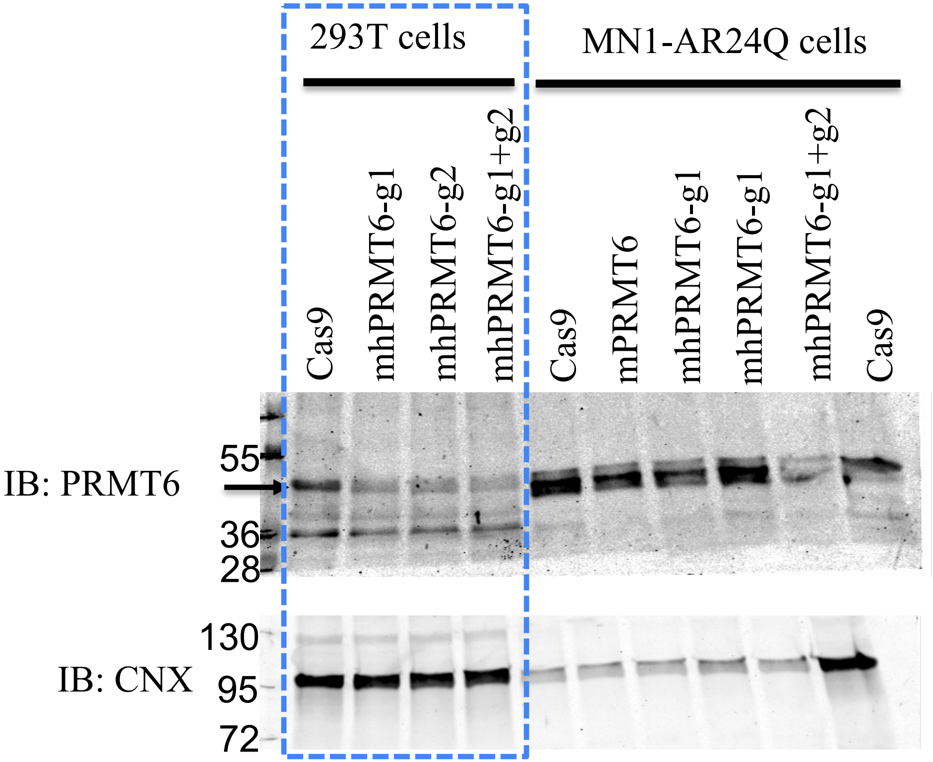

Uncropped blot for Figure 6a (left)

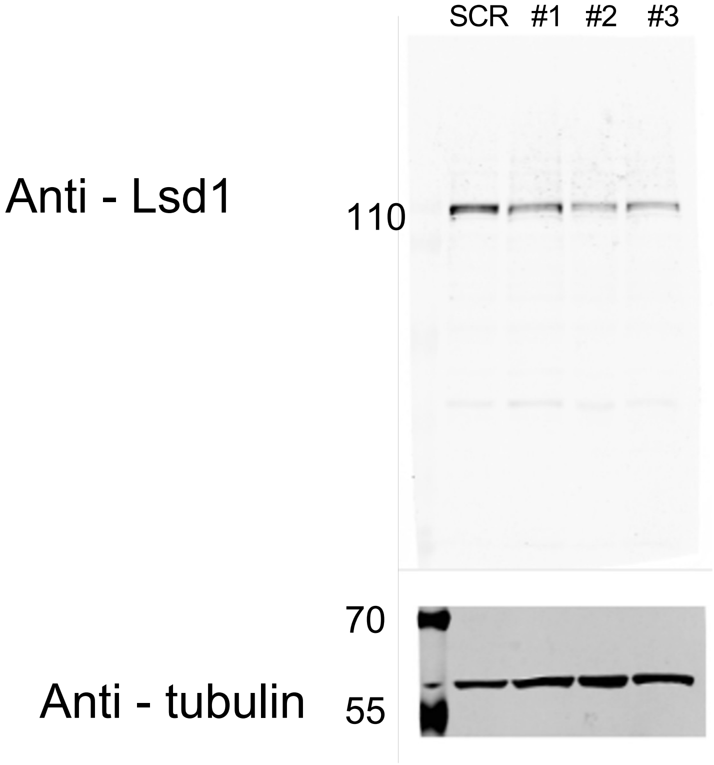

Uncropped fig. 6a (right)

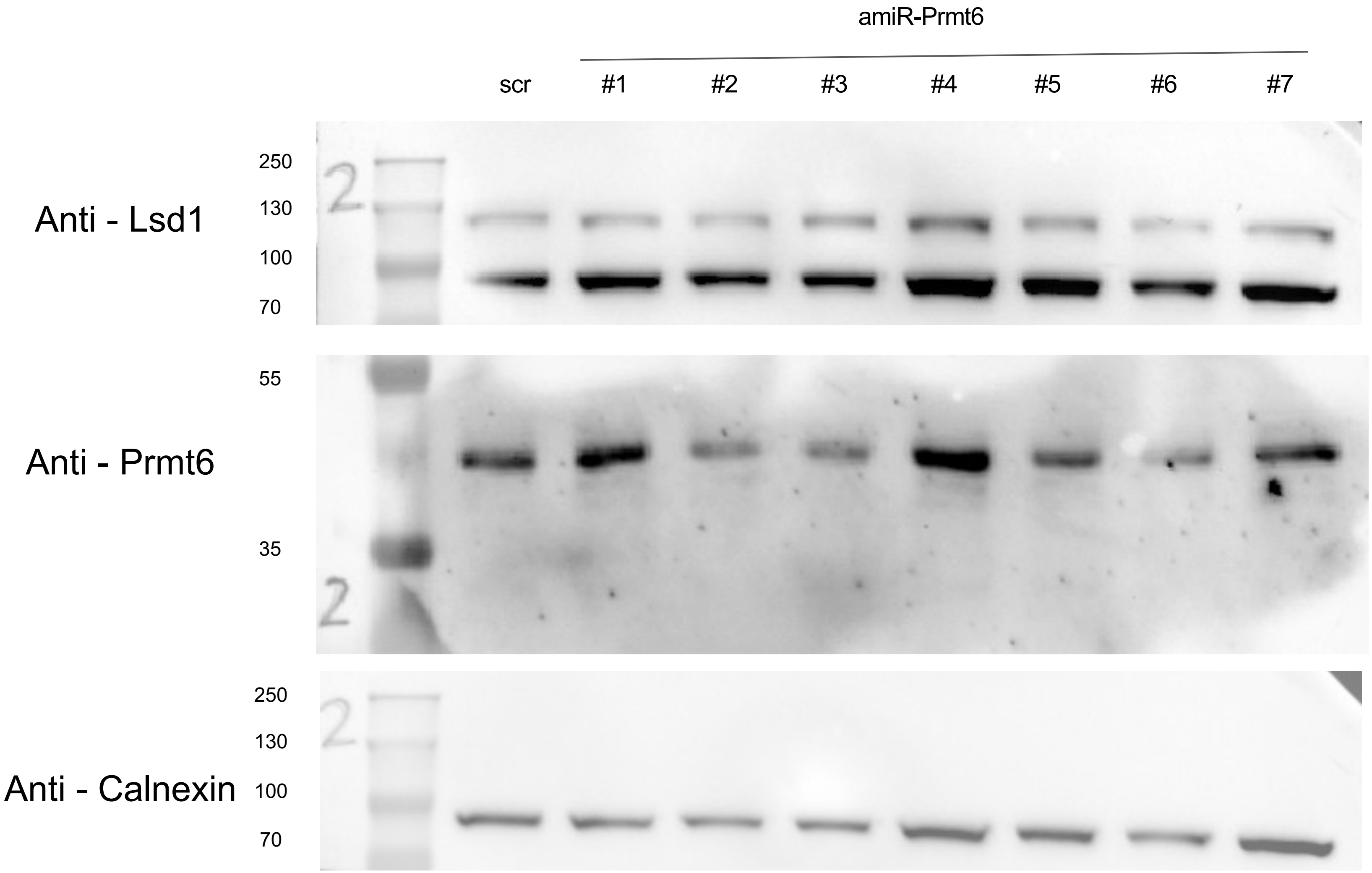

Uncropped fig.6e (2E10 virions)

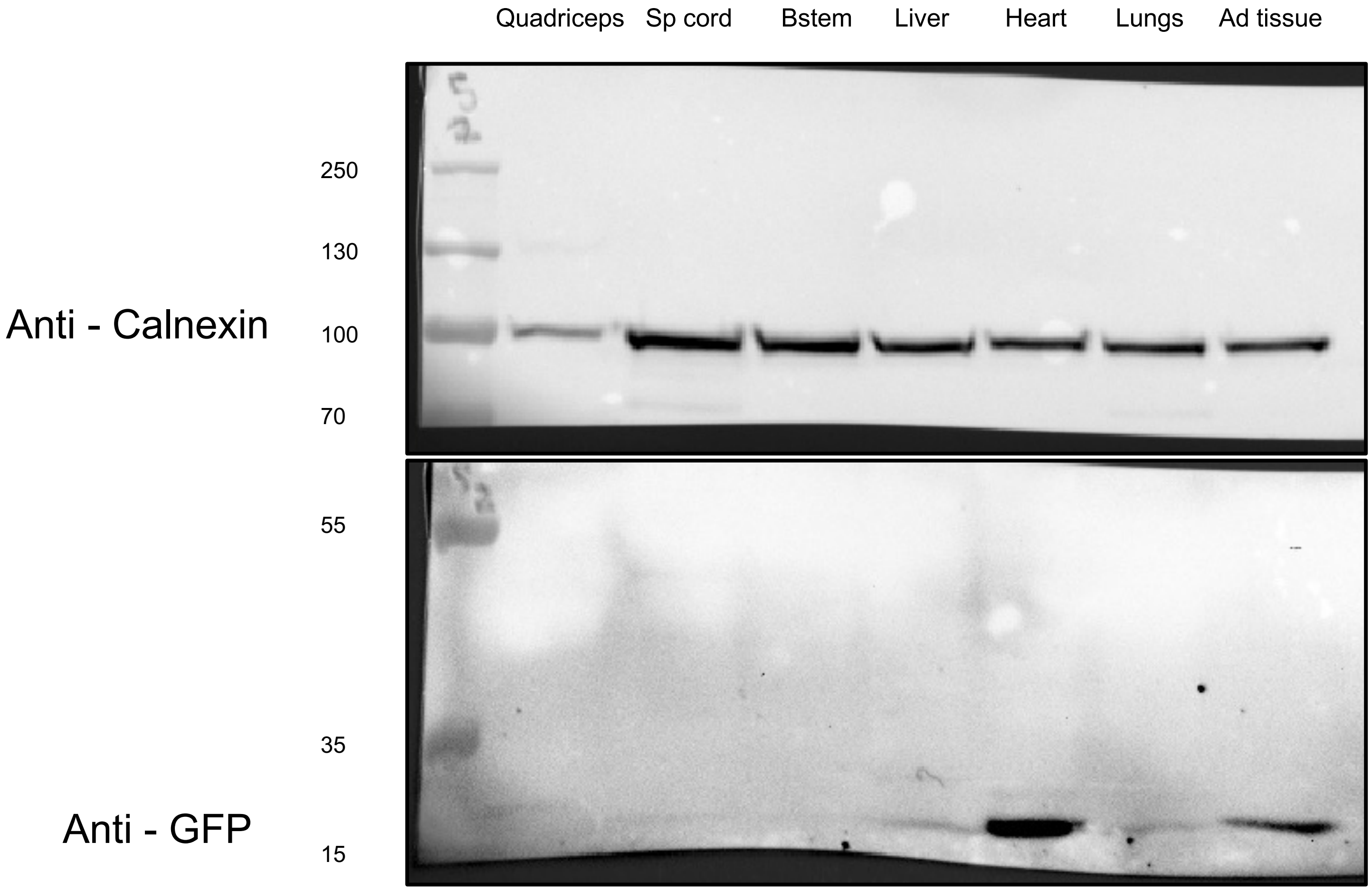

Uncropped fig.6e (8E10 virions)

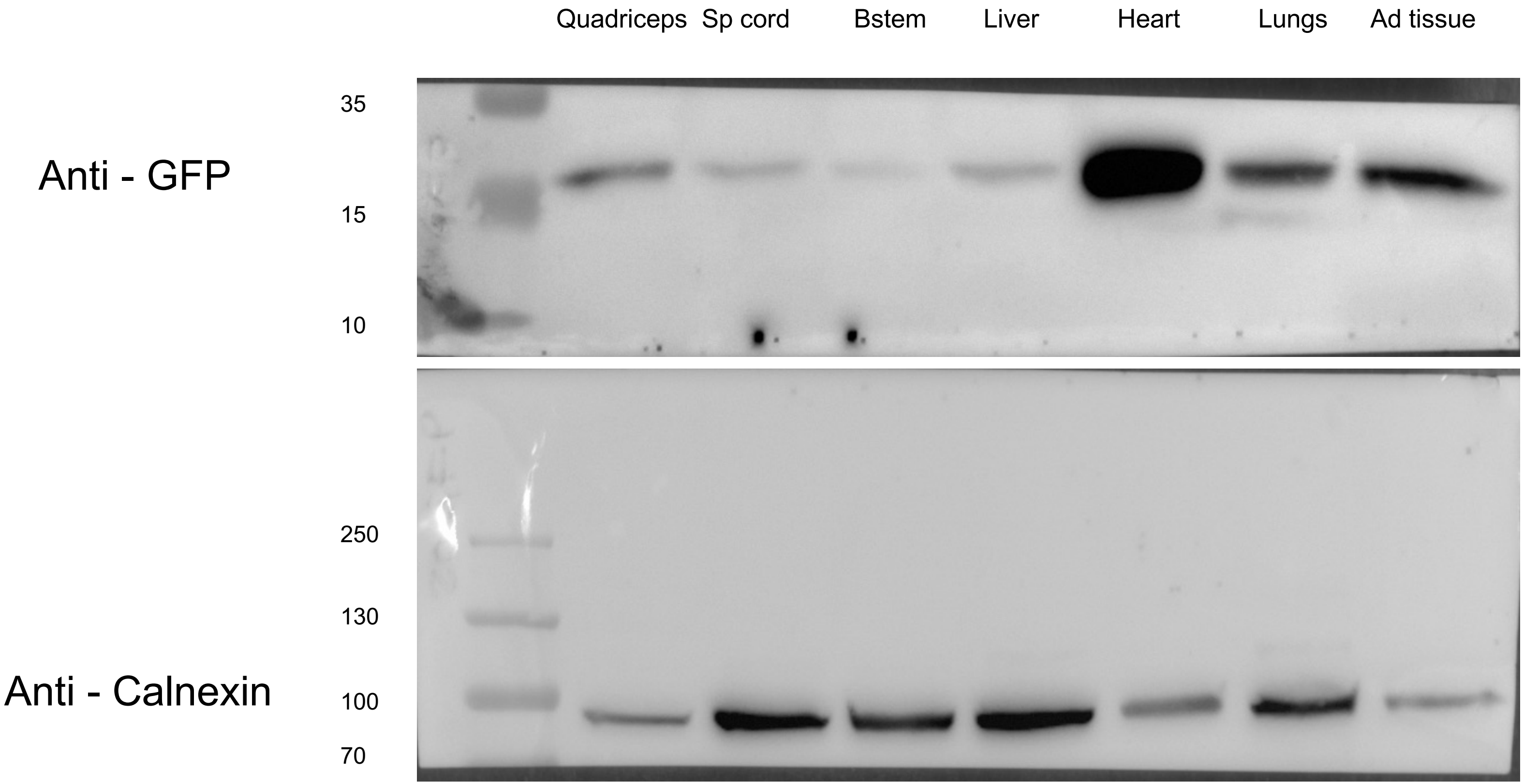

Uncropped fig.6g

IS: internal standard
P6KO: PRMT6 knockout mouse

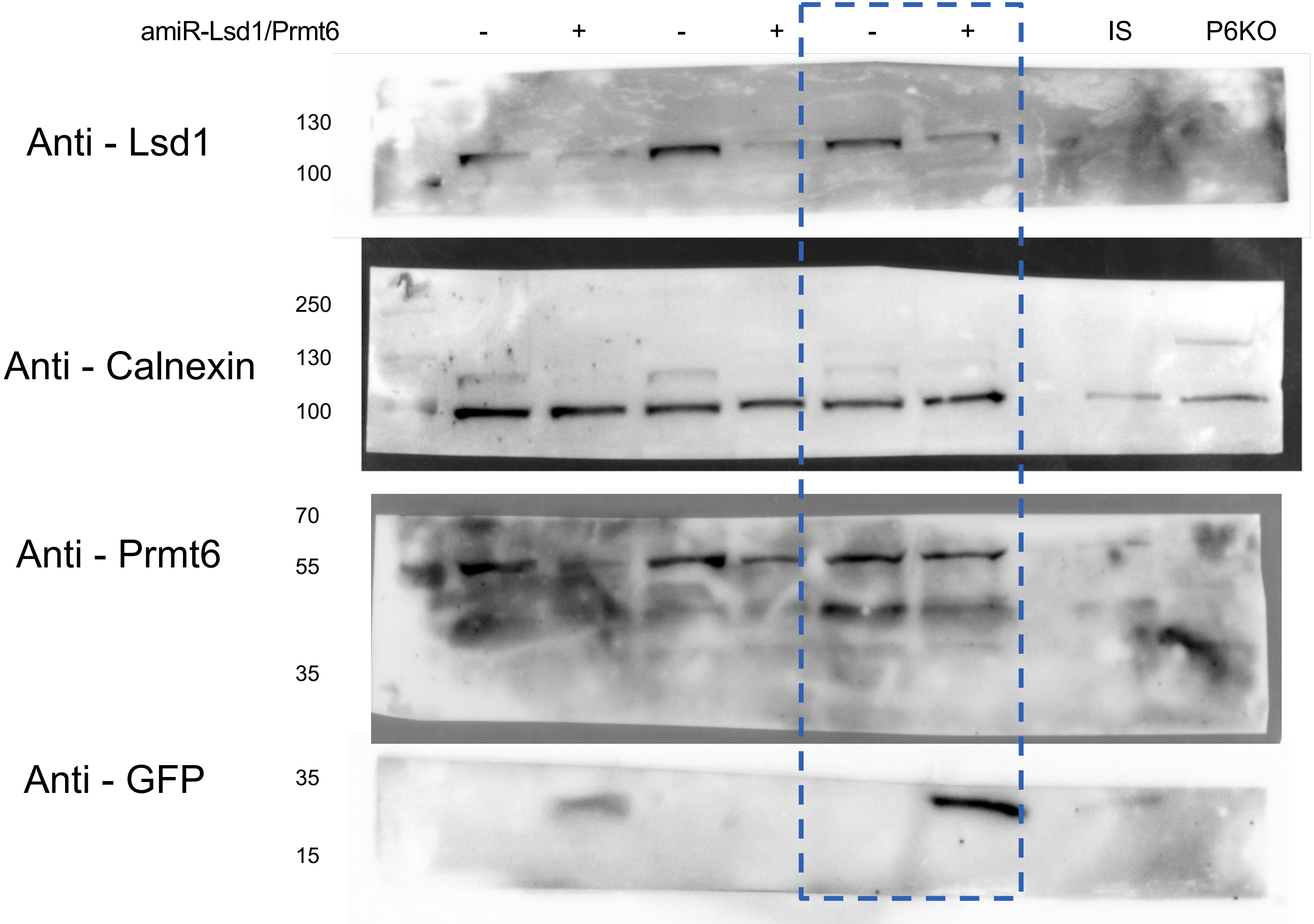

Uncropped fig. 8d AR mirna

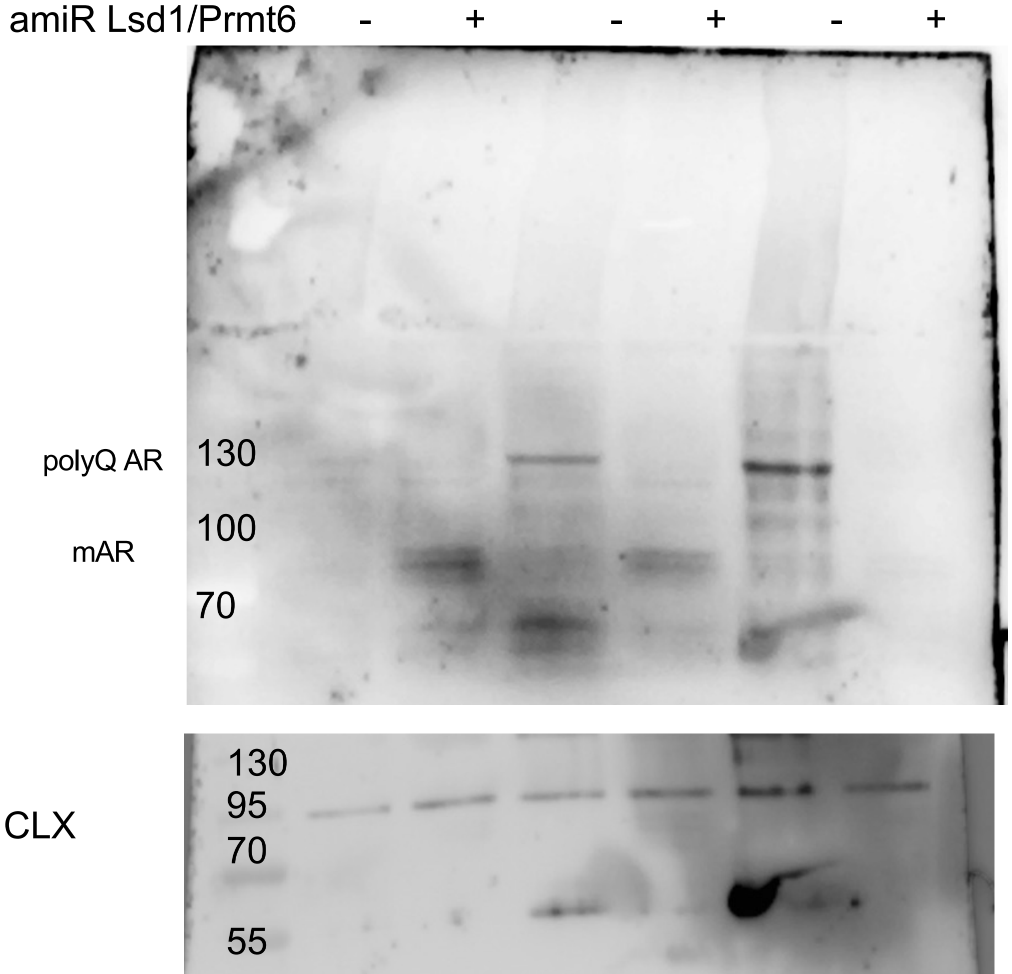

Uncropped blot for Figure 9a (left)

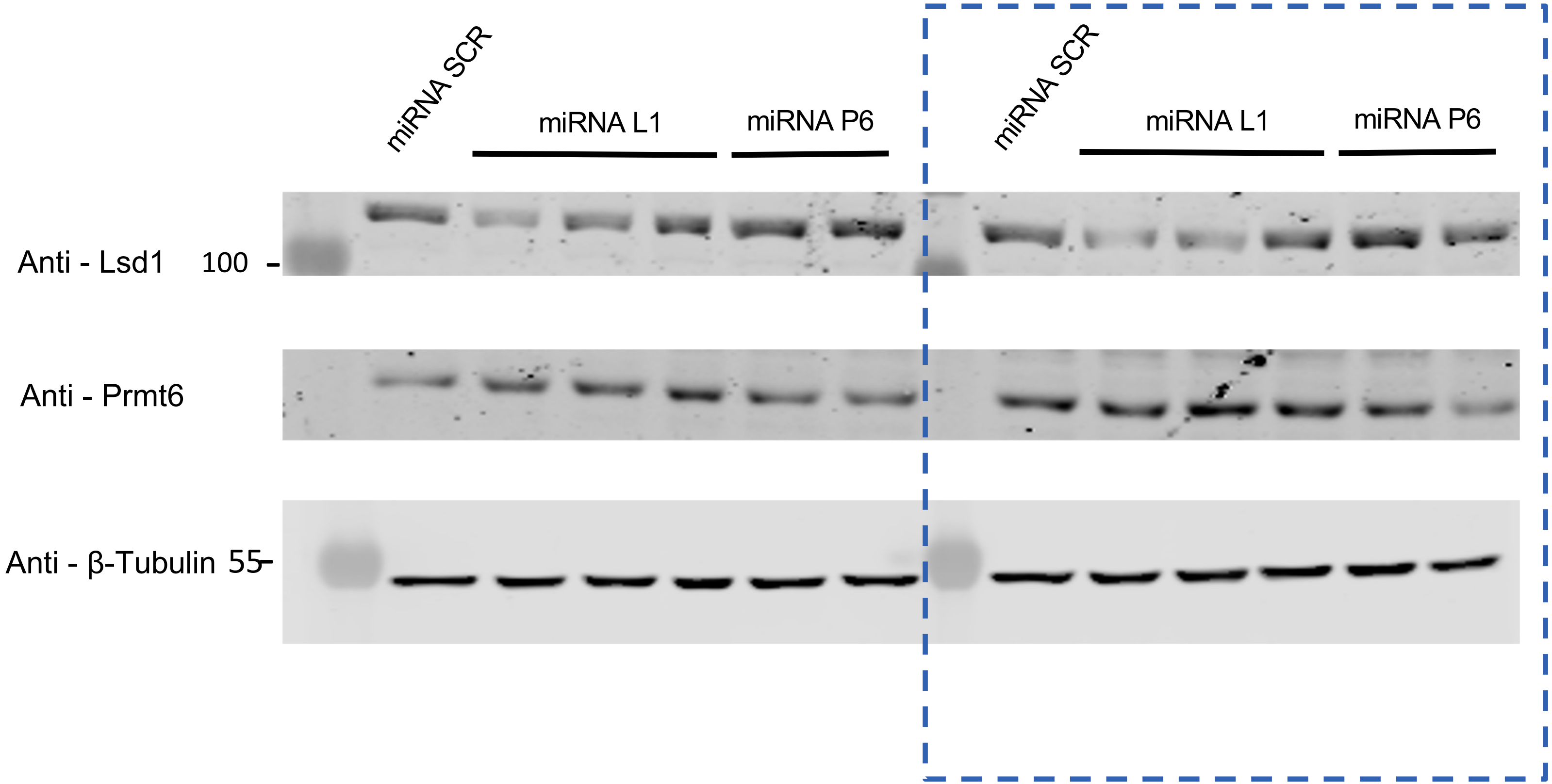

Uncropped fig. 9a (right)

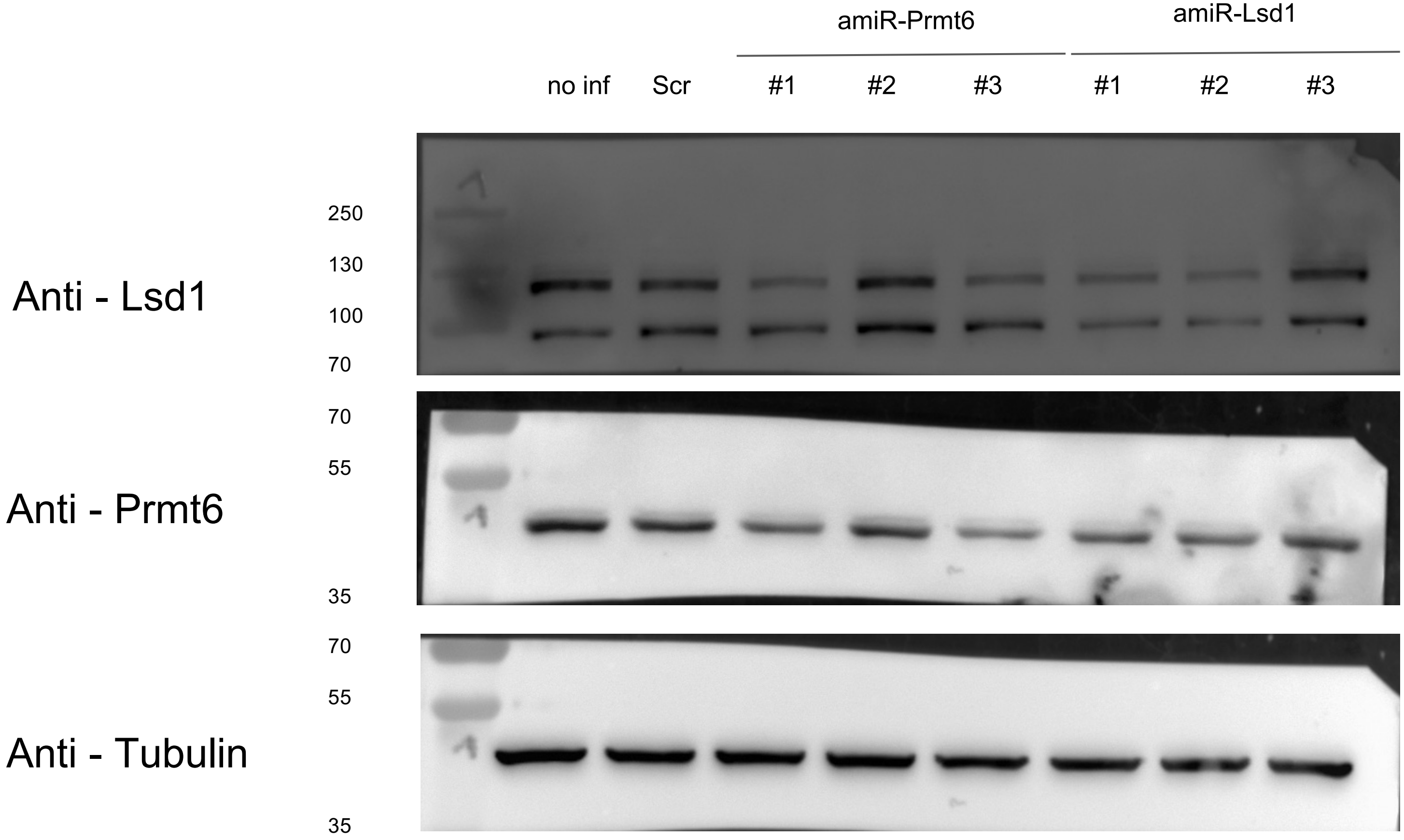

Uncropped Supp fig. 1a - Spinal cord

IS: internal standard

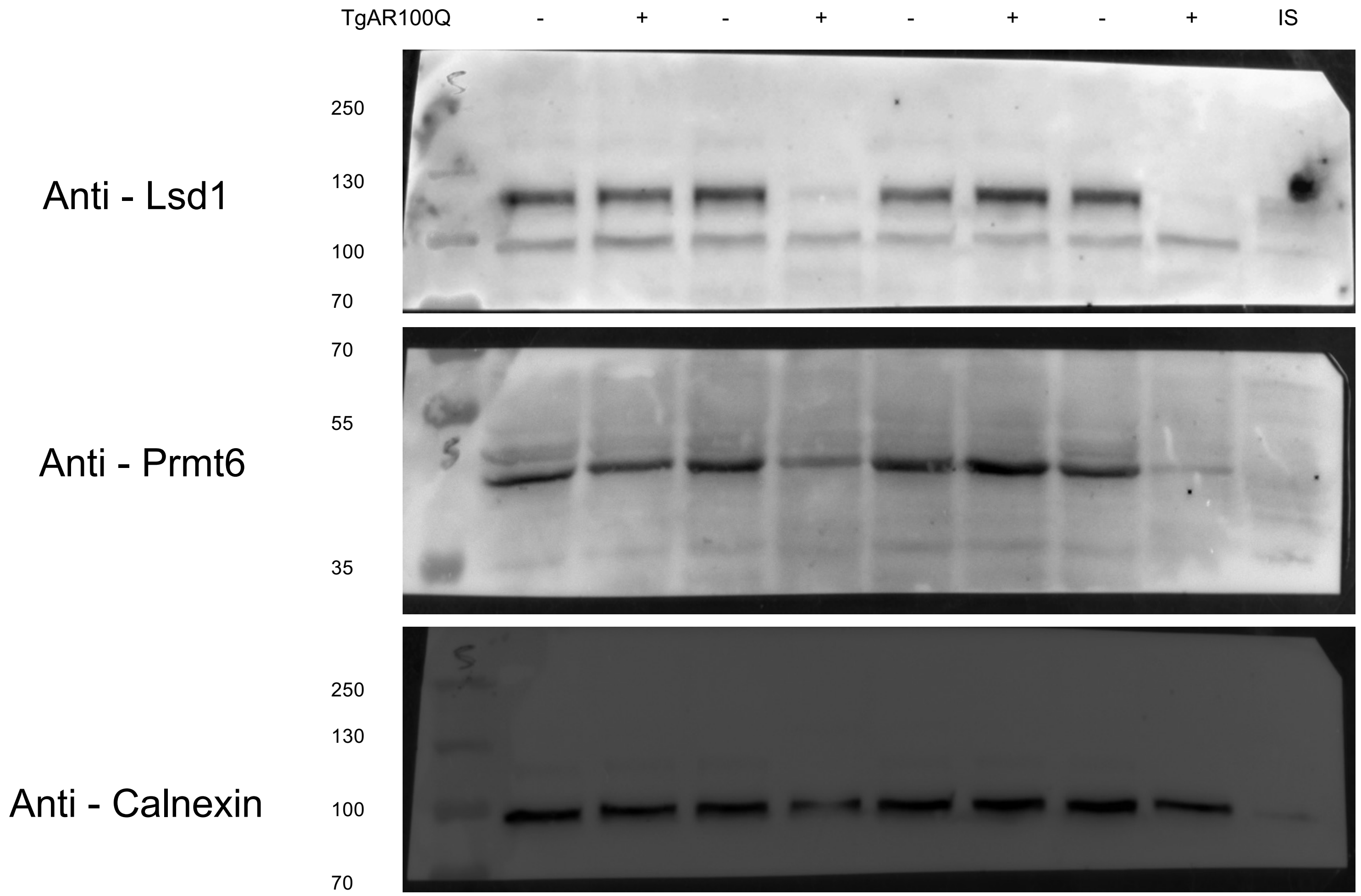

Uncropped blot for Supplementary figure 2a AR24Q/65Q IP with LSD1

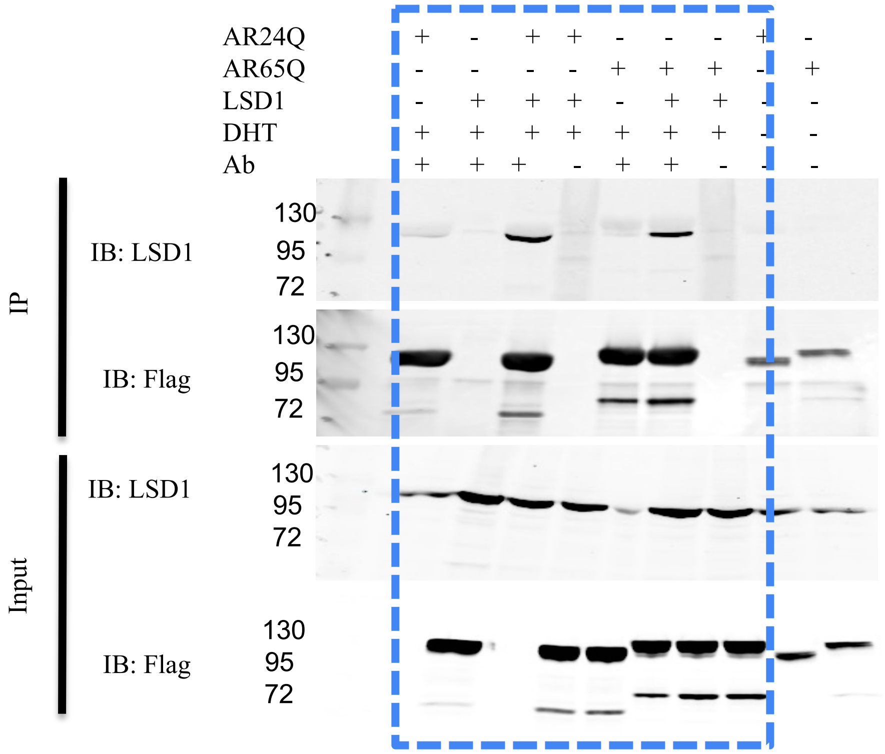

Uncropped blot for Supplementary Figure 2b

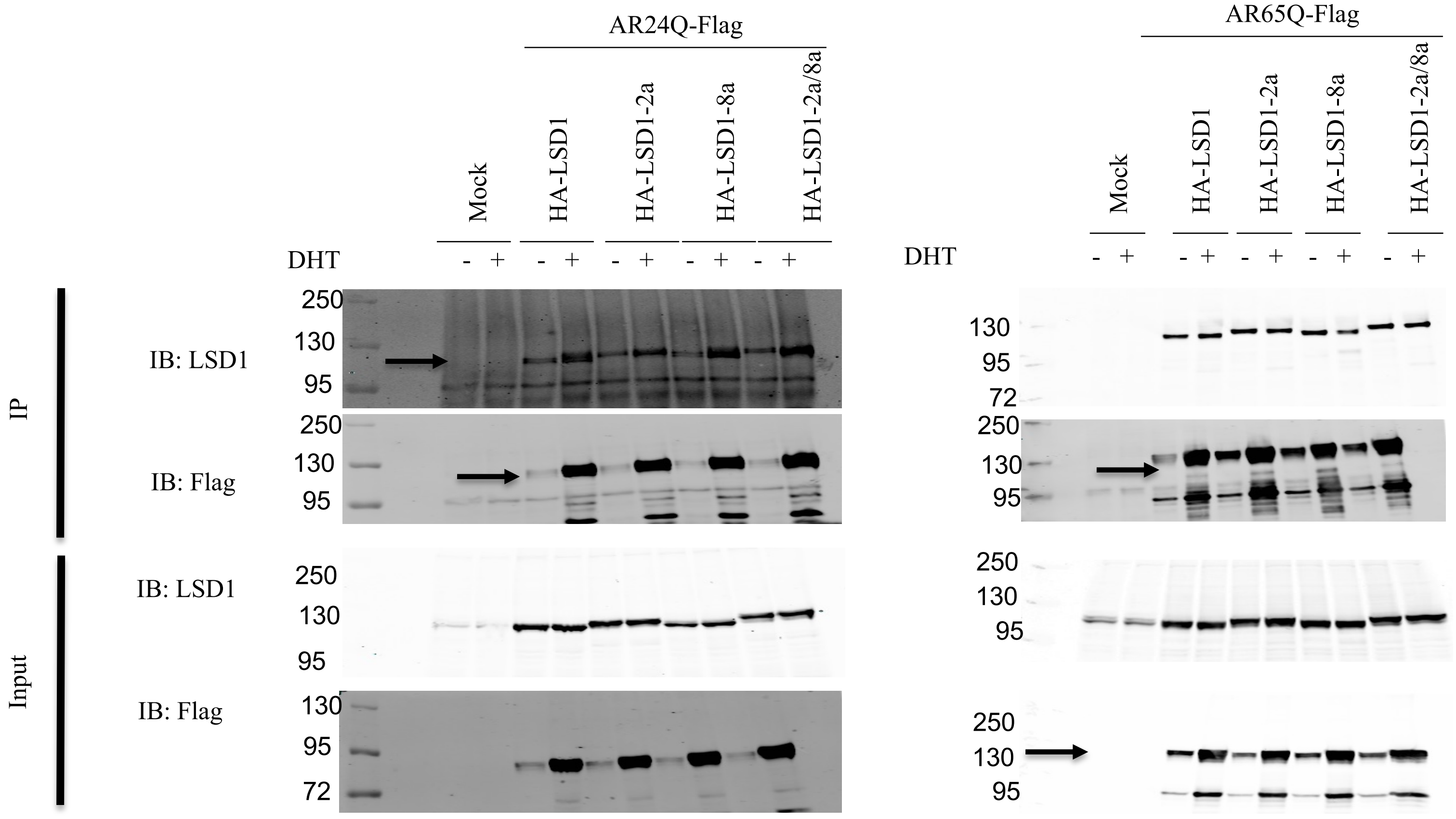

Supplementary figure 6a LSD1 and PRMT6 Co-IP in HEK293T cells

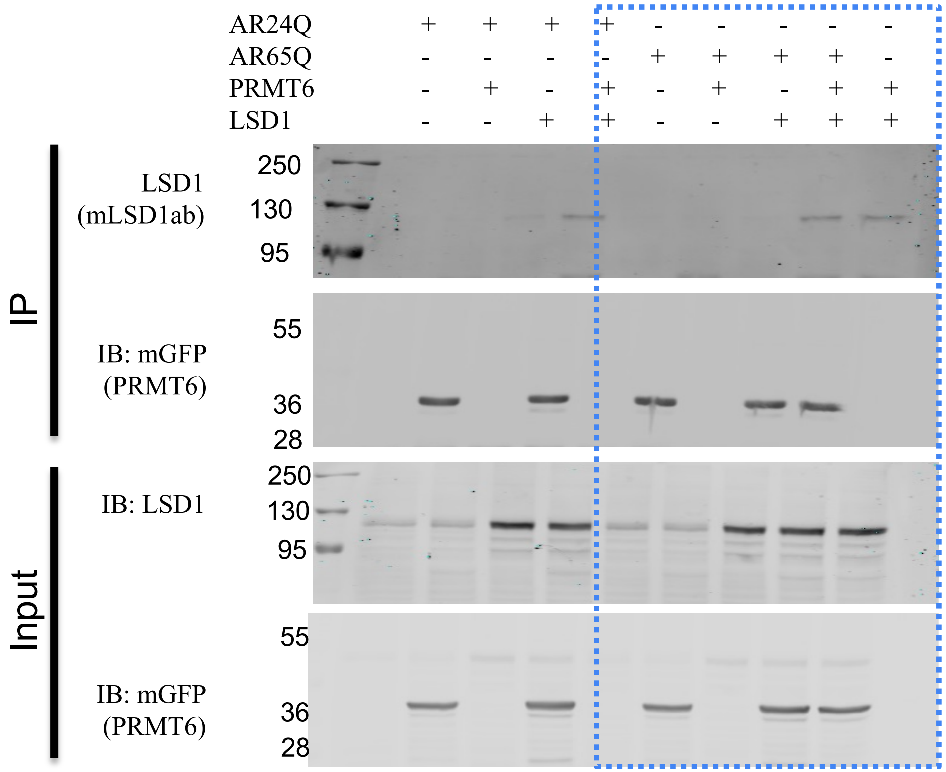

Uncropped blot for Supplementary Figure 6 A

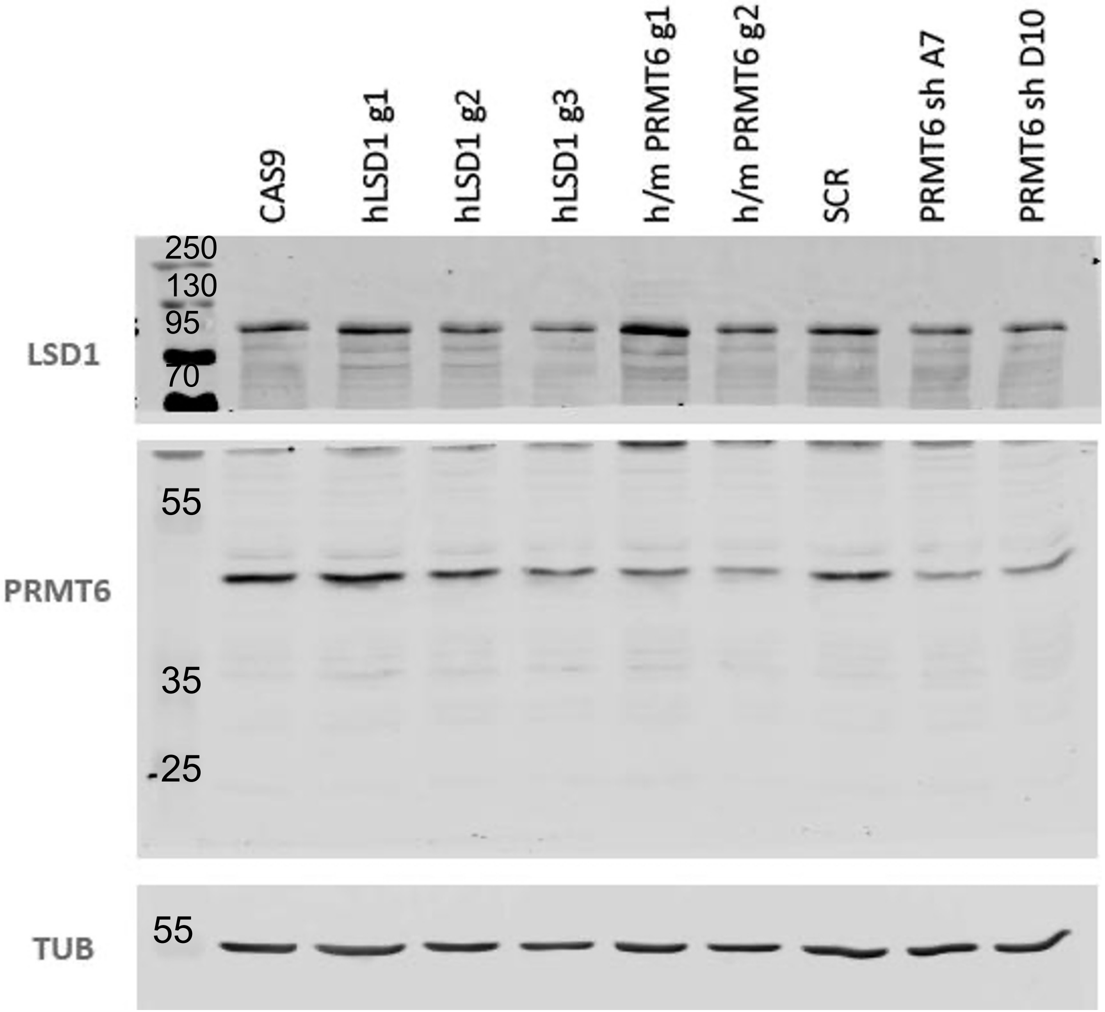

Uncropped Supp. fig 7

IS: internal standard
P6KO: PRMT6 knockout mouse

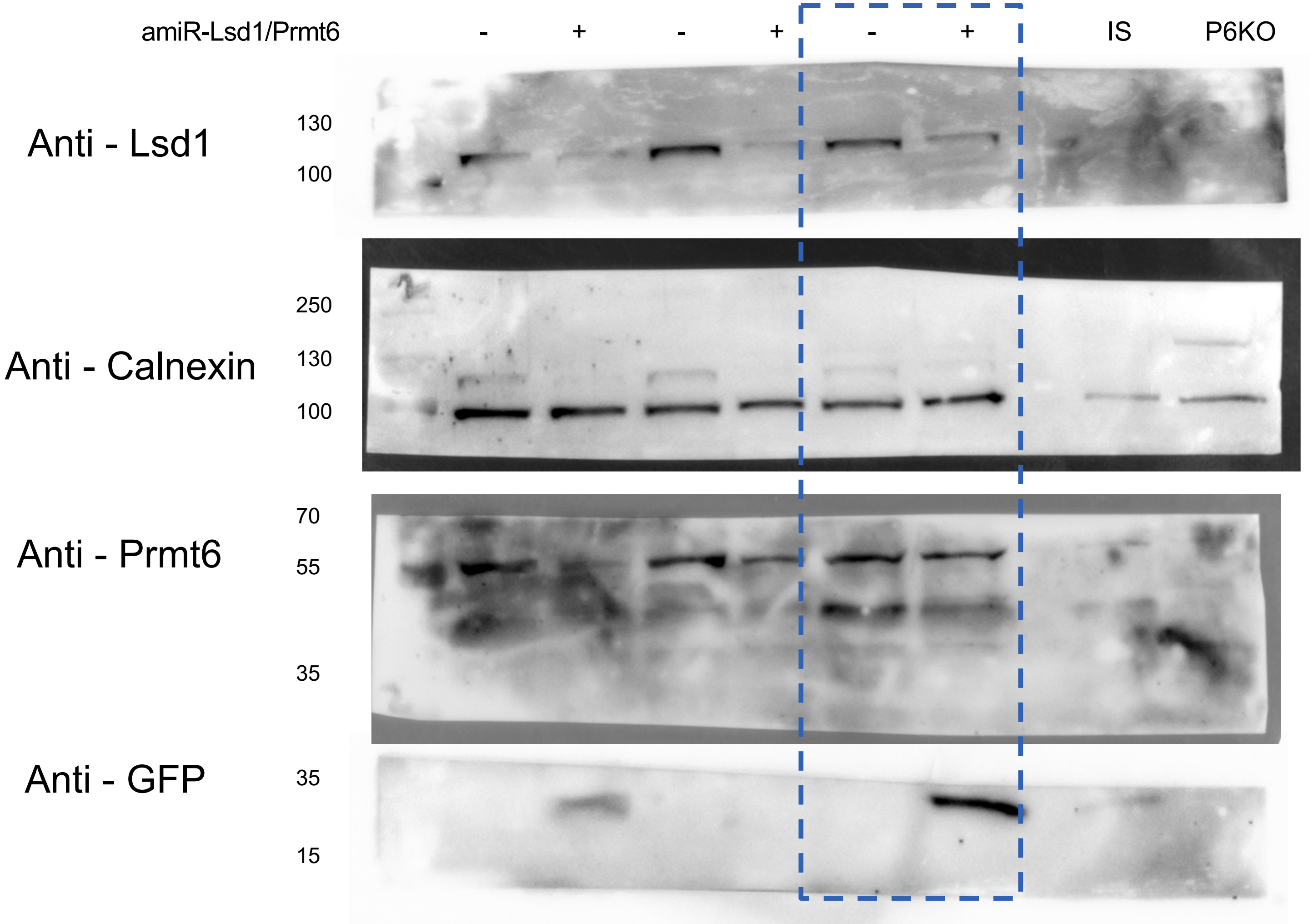

Uncropped Supp fig. 7 - Spinal cord

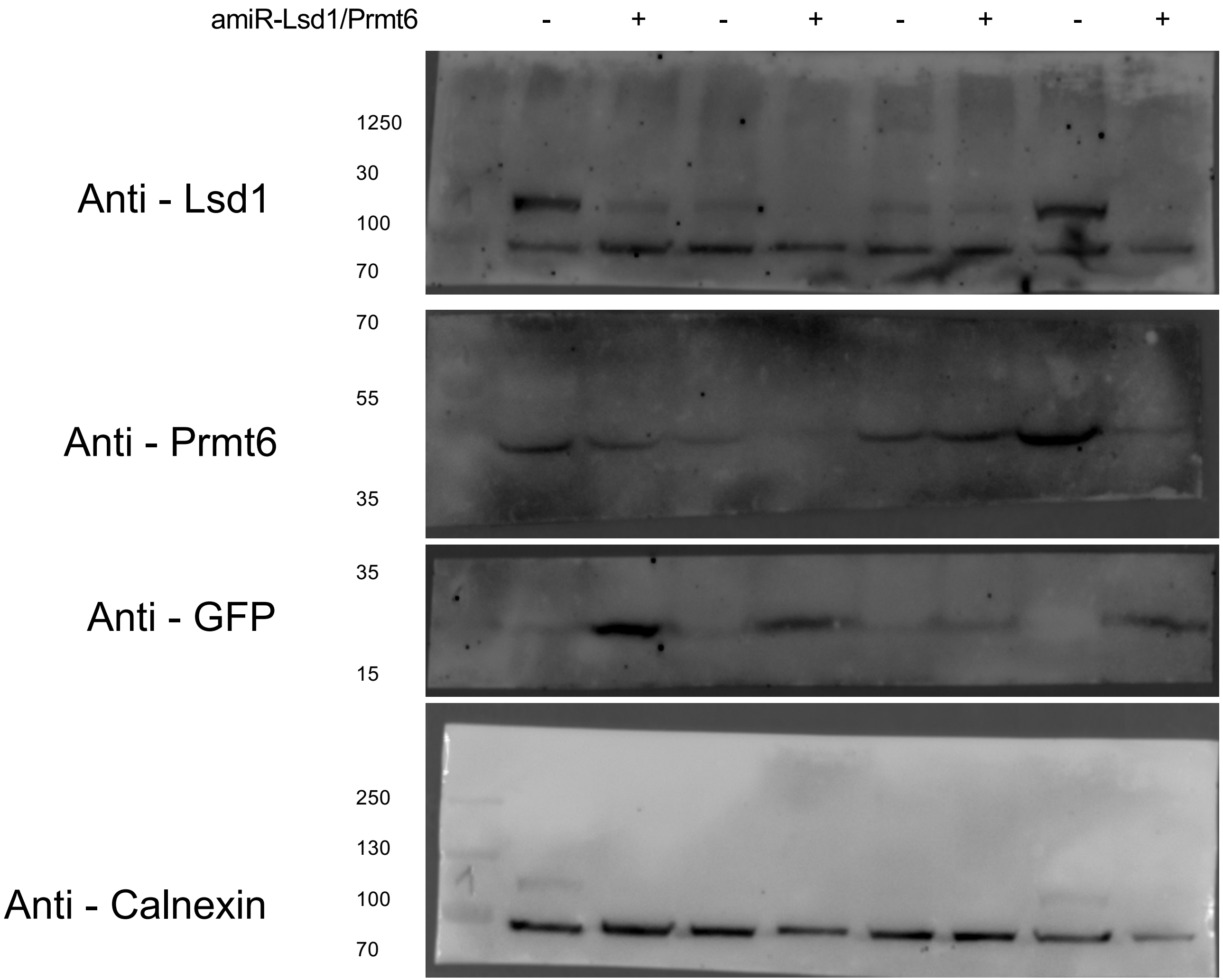

Uncropped Supp fig. 7 - Liver

IS: internal standard

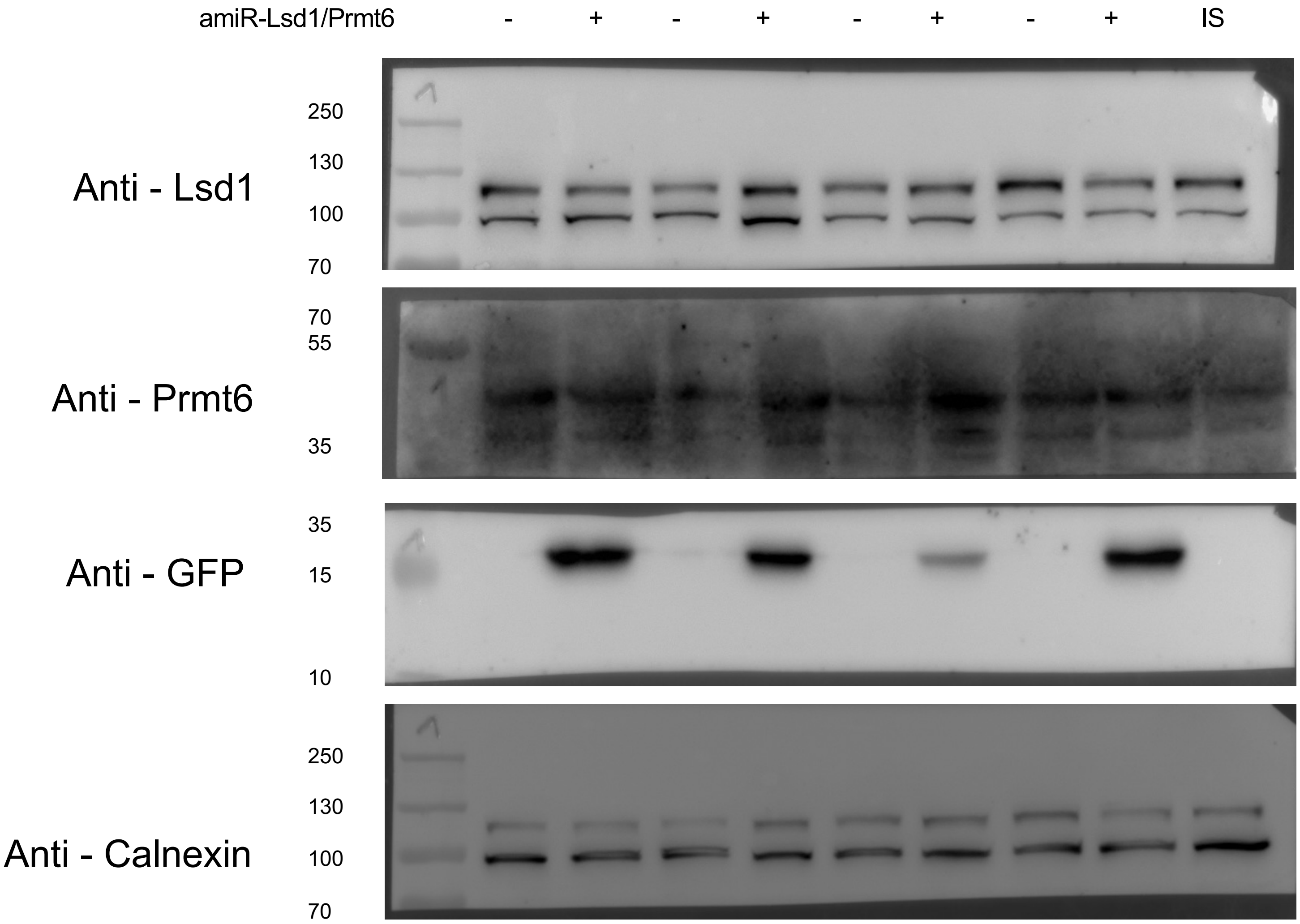

Uncropped Supp fig. 7 - Heart

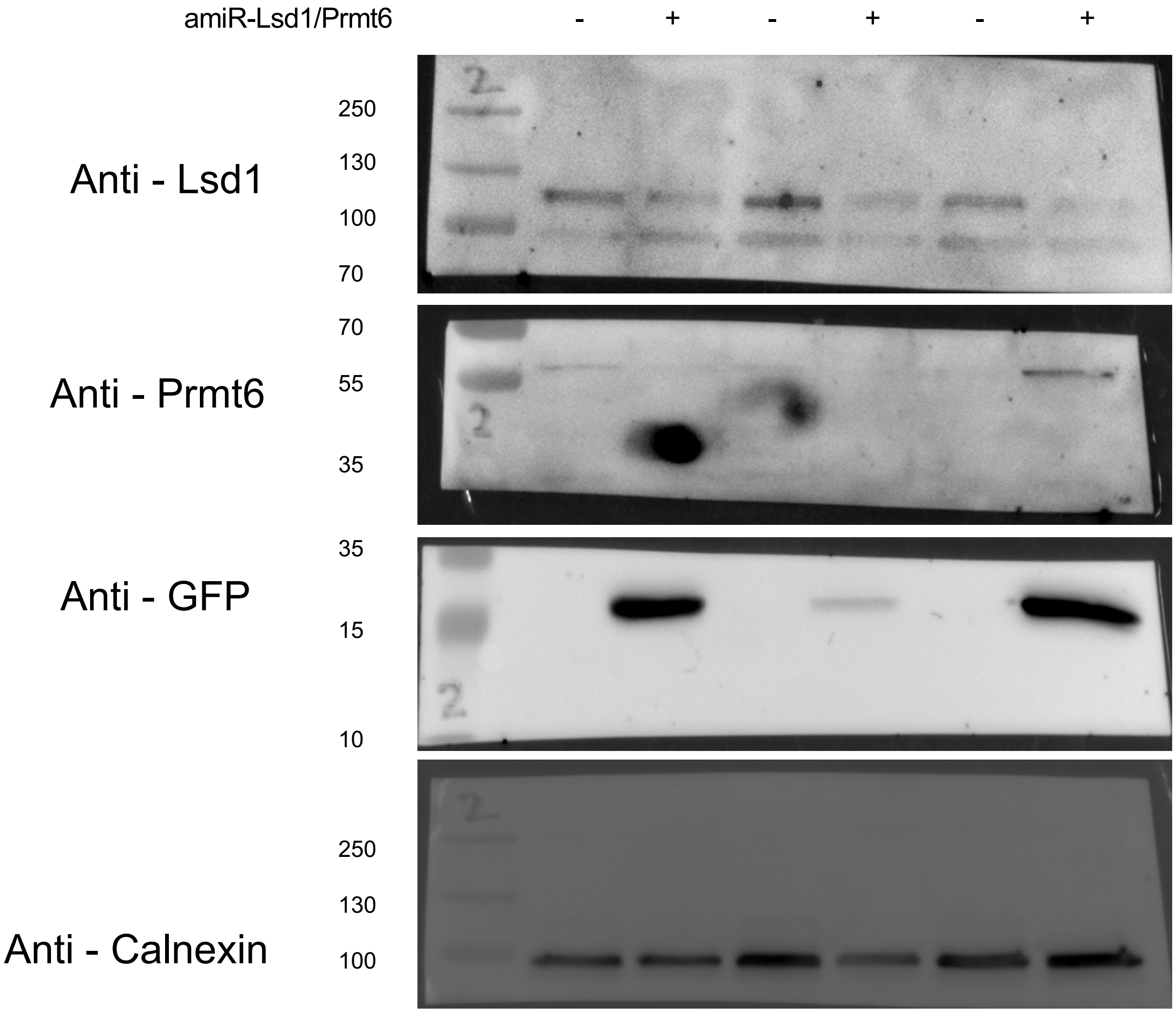

Uncropped blot for Supplementary Figure 7 – lungs

miRNA - + - + - + - +

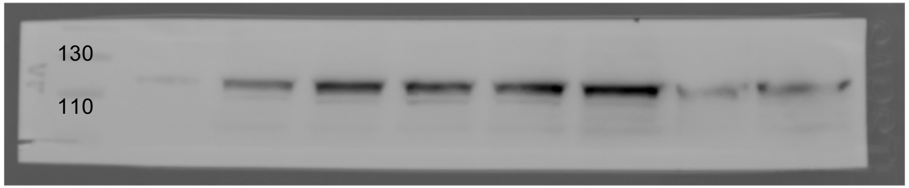

LSD1

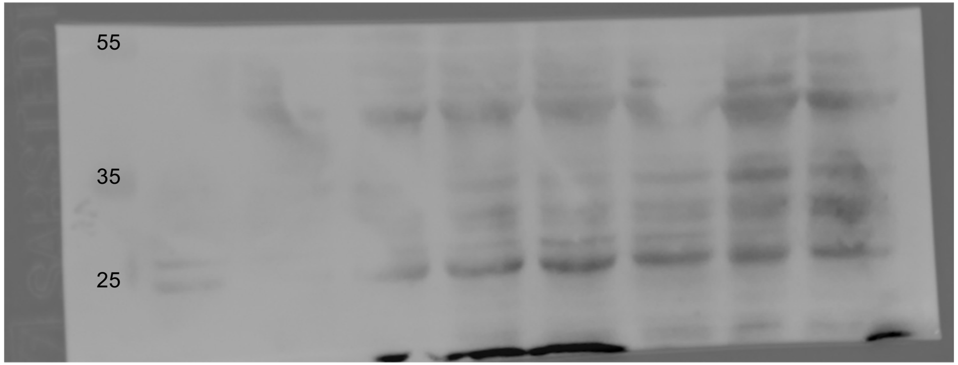

PRMT6

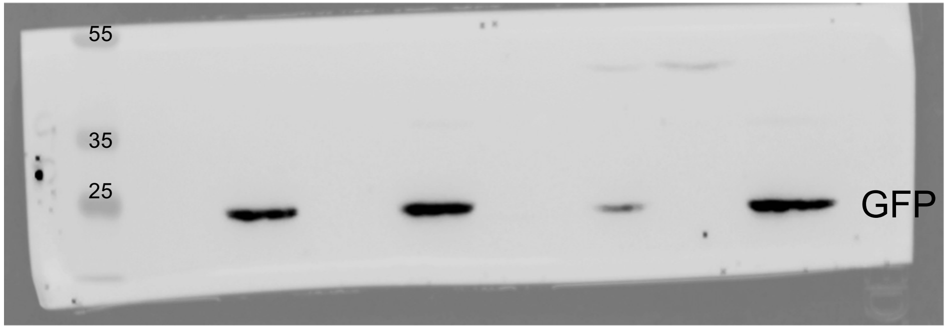

ACTIN

miRNA - + - + - + - +

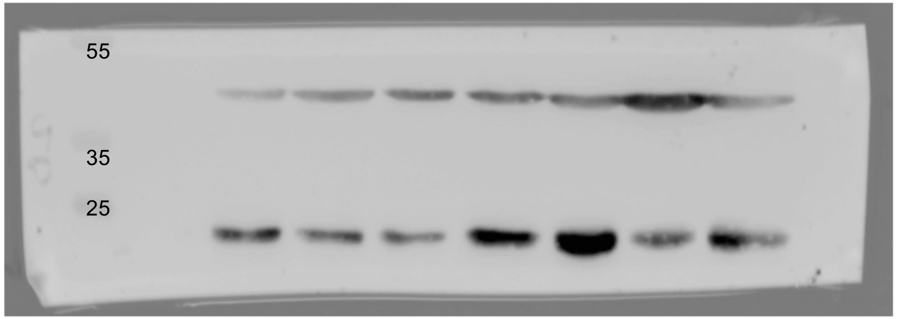

Uncropped blot for Supplementary Figure 7 – white adipose tissue

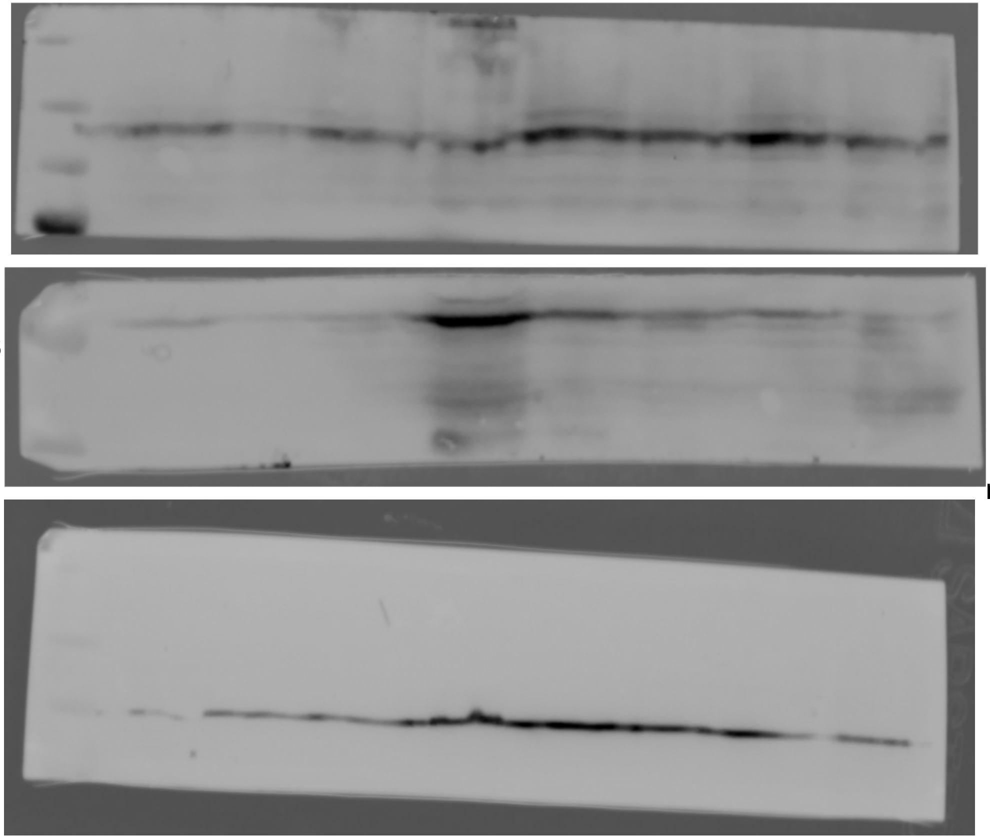

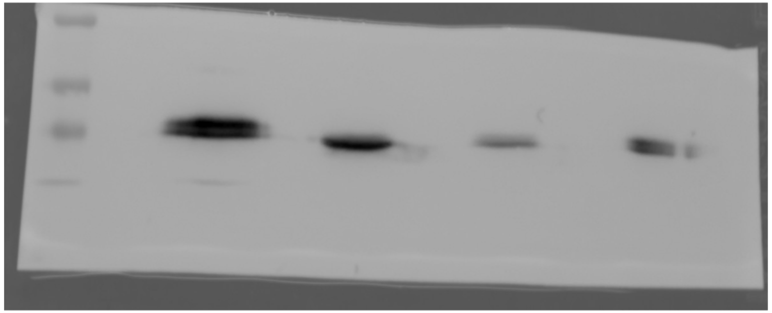

Supplement: Supplementary file 6 — Source Data [file 41467_2023_36186_MOESM6_ESM.zip › Uncropped figures_ final_rev 3.pdf]
